# Non-degradative Ubiquitination of Protein Kinases

**DOI:** 10.1371/journal.pcbi.1004898

**Published:** 2016-06-02

**Authors:** K. Aurelia Ball, Jeffrey R. Johnson, Mary K. Lewinski, John Guatelli, Erik Verschueren, Nevan J. Krogan, Matthew P. Jacobson

**Affiliations:** 1 Pharmaceutical Chemistry, University of California at San Francisco, San Francisco, California, United States of America; 2 Cellular and Molecular Pharmacology, University of California at San Francisco, San Francisco, California, United States of America; 3 Division of Infectious Diseases, University of California at San Diego School of Medicine, La Jolla, California, United States of America; Weizmann Institute of Science, ISRAEL

## Abstract

Growing evidence supports other regulatory roles for protein ubiquitination in addition to serving as a tag for proteasomal degradation. In contrast to other common post-translational modifications, such as phosphorylation, little is known about how non-degradative ubiquitination modulates protein structure, dynamics, and function. Due to the wealth of knowledge concerning protein kinase structure and regulation, we examined kinase ubiquitination using ubiquitin remnant immunoaffinity enrichment and quantitative mass spectrometry to identify ubiquitinated kinases and the sites of ubiquitination in Jurkat and HEK293 cells. We find that, unlike phosphorylation, ubiquitination most commonly occurs in structured domains, and on the kinase domain, ubiquitination is concentrated in regions known to be important for regulating activity. We hypothesized that ubiquitination, like other post-translational modifications, may alter the conformational equilibrium of the modified protein. We chose one human kinase, ZAP-70, to simulate using molecular dynamics with and without a monoubiquitin modification. In Jurkat cells, ZAP-70 is ubiquitinated at several sites that are not sensitive to proteasome inhibition and thus may have other regulatory roles. Our simulations show that ubiquitination influences the conformational ensemble of ZAP-70 in a site-dependent manner. When monoubiquitinated at K377, near the C-helix, the active conformation of the ZAP-70 C-helix is disrupted. In contrast, when monoubiquitinated at K476, near the kinase hinge region, an active-like ZAP-70 C-helix conformation is stabilized. These results lead to testable hypotheses that ubiquitination directly modulates kinase activity, and that ubiquitination is likely to alter structure, dynamics, and function in other protein classes as well.

## Author Summary

Attachment of ubiquitin to another protein is typically used to mark the protein for degradation by the proteasome. However, recent studies show that many proteins are tagged with ubiquitin and not degraded. We hypothesized that ubiquitin can regulate the protein it is attached to by changing its structure and dynamics. We performed proteomics experiments to identify all of the kinase proteins tagged by ubiquitin in a human cell line as well as the site of ubiquitination. We found that kinases are often ubiquitinated in structured regions important for regulation and activity. We then performed molecular dynamics simulations of one kinase, ZAP-70, to see if a ubiquitin tag could affect the kinase structure. We found that ubiquitin does affect the structure of ZAP-70, and the effect depends on where the ubiquitin is attached. At K377, ubiquitin changes the ZAP-70 structure to resemble the inactive state, while ubiquitin attached at K476, on the other side of the protein, has the opposite effect. These simulations indicate that ubiquitin, like other post-translational modifications, may alter the structure and dynamics of proteins in ways that impact activity and function.

## Introduction

The best-characterized function of ubiquitin is as a marker for protein degradation. The 76-residue ubiquitin protein can be covalently attached at its C-terminus to a lysine side chain amine group or N-terminus of another protein. Typically, additional ubiquitin molecules are also attached to the first ubiquitin, forming a polyubiquitin chain. The polyubiquitinated protein is then targeted to the proteasome for degradation, although ubiquitination can also result in lysosomal degradation [1–3]. Ubiquitin tagging for selective degradation is crucial to the regulation of many cellular processes [4].

Recently, alternate roles for ubiquitination that are not associated with degradation have gained attention. In 1992, Varshavsky *et al*. postulated that ubiquitin might have additional functions beyond proteasome targeting and that it might perturb the conformation of the proteins to which it is linked [5]. The residue linking two ubiquitin molecules along the polyubiquitin chain can influence whether the ubiquitinated protein is targeted for proteasomal degradation. The most common linkage, on K48, is closely associated with degradation by the proteasome [6]. K6, K11, K27, and K23 linkages have not been studied as extensively, but may also be associated with proteasomal degradation [7,8]. Levels of K63-linked ubiquitination, however, are not affected by proteasomal inhibition, suggesting alternate functions [6–8]. K63-linked polyubiquitination is also associated with protein trafficking, DNA repair, and inflammation [7]. Monoubiquitination also is not typically associated with degradation, and in some cases may regulate protein function by impeding or facilitating protein-protein association [8–10]. Finally, several ubiquitin-like proteins, such as NEDD8 and SUMO[3], are known to modify proteins in a similar manner to ubiquitin, and regulate function independent of degradation.

Several pathways have been identified in which ubiquitination influences function. One example is calmodulin, which is site-specifically and reversibly monoubiquitinated, reducing its activity toward phosphorylase kinase [11]. Non-degradative ubiquitination both positively and negatively regulates the TGF-β signaling pathway through mono and polyubiquitinated Smad proteins [12–14]. TAK1 polyubiquitination with a K63 linkage also regulates TGF-β signaling by activating TAK1 [15]. These ubiquitin modifications may modulate protein-protein interactions within the pathway, or directly induce conformational changes in the modified protein. A second example of non-degradative ubiquitination controlling function is the T cell receptor-zeta (TCR-zeta), which is polyubiquitinated by Cbl-b and Itch with a K33 linkage. This modification regulates TCR-zeta phosphorylation and its association with protein kinase ZAP-70 in a non-proteolytic manner [16–18]. Rather than inducing degradation, the ubiquitin chain promotes phosphorylation, possibly by interacting with the kinase Lck, or by altering the conformation of the TCR-zeta.

There are several possible mechanisms for how ubiquitin might regulate protein function independent of degradation. Computational structural modeling suggests that ubiquitination can directly affect protein function by blocking a functional domain or active site, sterically preventing oligomerization, preventing target protein binding, or restricting protein flexibility [19]. A perspective by Chernorudskiy and Gainullin details examples of ubiquitination that regulates protein structure and function without involvement of a ubiquitin binding domain (UBD) [20]. Monoubiquitination of histone H2B has a steric effect, preventing chromatin compaction which allows increased methylation of histone H3, and does not depend on recognition by a UBD [21,22]. Ras is another example of activation by site specific monoubiquitination [23]. Different Ras GTPase isoforms are monoubiquitinated at distinct sites which result in distinct mechanisms of Ras activation, and chemical attachment of monoubiquitin at these sites recapitulates this result [24,25]. This suggests a conformational change that is different depending on the ubiquitination site. Alpha-synuclein also shows a conformational dependence on ubiquitination site in terms of propensity to form fibrils and oligomers [26]. Certain ubiquitination sites on alpha-synuclein result in proteasomal degradation, while other sites do not [27].

Crystal structures of these ubiquitinated proteins would be very useful for determining what conformational changes occur upon ubiquitination and how these affect function. Two crystal structures of monoubiquitinated constructs have been solved for proliferating cell nuclear antigen (PCNA) [28,29]. These structures show that the ubiquitin is flexible, interacting with PCNA through the canonical hydrophobic patch at I44 or extended away from PCNA due to electrostatic repulsion, which is consistent with SAXS data [29,30]. The structures show changes in PCNA conformation upon ubiquitination, and mutational studies indicate that the ubiquitin hydrophobic patch can directly interact with the polymerase, facilitating polymerase exchange. The ubiquitin moiety also provides a steric restriction on proteins interacting with PCNA. A recent combined SAXS and computational study shows that SUMOylation has a different effect on PCNA conformation than ubiquitination [31]. A crystal structure of SUMOylated thymine DNA glycosylase (TDG) also reveals a conformational change in the modified protein caused by attachment of the SUMO moiety [32]. SUMOylation of the TDG C-terminal domain results in formation of a helix at the TDG-SUMO interface that disrupts TDG function [32], a mechanism which could also occur in ubiquitination. Crystal structures of ubiquitin conjugated to an E2 ubiquitin conjugating enzyme [33] and a HECT E3 ubiquitin ligase [34,35], reveal that a ubiquitinated protein can form a compact interaction with ubiquitin with the canonical hydrophobic patch either buried or solvent exposed.

Although no crystal structures of a polyubiquitinated target protein are available, many structural studies have been performed on polyubiquiitn chains on their own, which have implications for the biophysical consequences of polyubiquitination. K48-linked diubiquitin and tetraubiquitin both adopt compact, globular conformations with the ubiquitin moieties bound together, burying the I44 hydrophobic patch at the interdomain interface [36–38]. However, other linkages form different conformations as a polyubiquitin chain. K63-linked diubiquitin adopts an open, extended conformation, and as a tetrameric chain it fluctuates between extended and compact conformations [39–44]. The rarer linkages have not been studied as thourghly, but K33-linked, K29-linked and linear diubiquitin are observed to adopt extended structures without significant contacts between the ubiquitin monomers, similarly to K63 [39,45]. In crystal structures, K11 and K6-linked diubiquitin chains form compact conformations that are globular, but distinct from K48-linked diubiquitin [46,47]. From these polyubiquitin structures, it seems likely that K48-linked polyubiquitin would have a different physical effect on its target than K63-linked polyubiquitin or one of the other linkages that adopts an extended conformation. In an extended structure, it is possible that only the proximal ubiquitin domain in the polyubiquitin chain would directly interact with the target protein.

In this work, we focus on ubiquitin modifications of protein kinases, because many mechanisms for kinase regulation have been characterized previously. The role of post-translational modifications (PTM), especially phosphorylation, in regulating kinase activity has been extensively investigated. A common site of kinase phosphorylation is the activation loop, where the introduction of the negatively charged phosphate is usually critical to complete activation [48]. Other examples of kinase regulation induced by phosphorylation include activation by releasing an autoinhibitory element (PKA, PKC, EphB2 and TGF-β receptor tyrosine kinases) [48–50], inactivation by sterically hindering access to the active site (CDKs) [51], inactivation by triggering association with an autoinhibitory domain (Src kinases) [48,52], and inactivation by inhibiting dimerization (DAPK2) [53]. Phosphorylation at one site can also affect phosphorylation at another site, as in the case of AMPKα, where phosphorylation at S485 or S173 reduces phosphorylation of the activation loop (T172) [54]. Beyond phosphorylation, lysine acetylation can also play a role in kinase regulation. For example, AMPK is inhibited by acetylation of a regulatory subunit [54,55].

Studies of kinase regulation by ubiquitination have demonstrated decreased kinase activity by triggering proteasomal or lysosomal degradation, as in the case of tyrosine kinase cell surface receptors [56] and AMPK [54]. Ubiquitination of Sic1 activates the cyclin-CDk protein complex, when Sic1 dissociates from the complex before being degraded by the proteasome [57]. However, there are also indications that ubiquitin and ubiquitin-like proteins can regulate kinases in a degradation-independent manner. In T cells, Cbl-b ubiquitinates and negatively regulates PI3-kinase, and this is not associated with degradation [56]. SUMOylation was found to increase activity of AMPK-β by increasing activation loop phosphorylation [54].

Recent developments in mass spectrometry and antibody enrichment of samples for ubiquitinated proteins have enabled several cell-wide proteomics studies of ubiquitinated proteins [8,58–61]. These experiments identify the precise site of ubiquitination on the protein and can indicate whether that ubiquitination site is associated with proteasomal degradation; however, they lack the ability to distinguish between mono and polyubiquitination. Kim *et al*. found that 42% of the ubiquitination sites in their study did not increase by more than 2-fold when the proteasome was inhibited, indicating that ubiquitination at these sites may have a function other than proteasomal degradation. In particular, they observed that when ubiquitin itself was ubiquitinated at K63 (indicating K63-linked polyubiquitination of an unknown target), this was not associated with proteasomal degradation [8].

In this study, we report proteomics data on ubiquitination of 132 kinases in Jurkat and cells, both in the presence and absence of the proteasome inhibitor MG132. We identify many ubiquitination sites on protein kinases that do not increase substantially upon treatment with MG132. Combining our data with previously published data from the PTMfunc database [60], we find 107 kinase ubiquitin modifications that are proteasome sensitive, and 103 that are proteasome insensitive. This suggests that ubiquitination of some kinases likely has a downstream effect other than proteasomal degradation. The sites of ubiquitination are most commonly found within folded protein domains, as has been observed of ubiquitination generally [60,62–64]. In the kinase domains, these sites are also concentrated in regions known to be important for regulating activity.

We hypothesized that ubiquitination, like other PTMs such as phosphorylation and acetylation, modulates the conformation and dynamics of the protein to which they are attached. Molecular dynamics (MD) simulations have been used to study the ways in which phosphorylation changes protein energy landscapes, and to thereby obtain new insights into the mechanisms by which phosphorylation affects protein function [65–69]. The primary effect of phosphorylation on the energy landscape is often electrostatic because the phosphate group introduces a -2 charge, which can disrupt hydrogen-bonding networks or introduce new stabilizing interactions. Using MD, lysine acetylation has also been shown to have allosteric effects on protein conformation and influence conformational stability [70,71].

Ubiquitin differs from these well-characterized modifications in that it is much larger and attached via a short but flexible linker, and it can be extended as a polyubiquitin chain with different linkage types. To begin exploring the ways in which ubiquitin modulates protein structure and dynamics, we performed a series of MD simulations for one kinase from our proteomics data, ZAP-70 tyrosine kinase, because it is ubiquitinated at several sites that are proteasome insensitive. ZAP-70 is a member of the Syk gene family of protein tyrosine kinases involved in T cell development and activation [72]. It consists of a kinase domain and two SH2 domains, which bind to the hinge region of the kinase, trapping it in an inactive conformation [73–77]. This autoinhibition is released when the SH2 domains bind to the phosphorylated form of the TCR complex ITAMs [75,76,78–80]. The SH2 domains detach from the kinase domain, exposing two conserved tyrosine residues on the linker between the SH2 and kinase domains, which are subsequently phosphorylated [72,73,75–77]. The crystal structure of the unphosphorylated ZAP-70 kinase domain without the SH2 domains bound adopts an active-like conformation [76,81]; however, phosphorylation on Y493 on the activation loop is required for full activation [72,75,76]. The downstream effect of ZAP-70 activation is Cbl phosphorylation and ultimately T cell activation [72]. Although a few studies have noted that ZAP-70 can be ubiquitinated *in vivo* [18,82,83], no one has examined how ubiquitination affects ZAP-70 conformation or activity. We performed MD simulations of two different monoubiquitinated constructs of ZAP-70 and characterized the sampled conformational ensembles. The proteomics data does not determine the number of ubiquitin molecules, so we attached only a single ubiquitin molecule, as this is the minimal possible modification and may have an effect that is relavent to all types of ubiquitination. These were compared to simulations of the unmodified ZAP-70 as well as other (non-ubiquitin) modifications to the same sites. The results demonstrate that ZAP-70 ubiquitination modulates structure and dynamics in a site-specific manner, and in ways that suggest possible regulation of kinase activity.

## Methods

### Ubiquitination and protein abundance proteomics experiments

For ubiquitination analysis in Jurkat cells, cells were first SILAC-labeled for at least 10 doublings in either “light” media containing standard amino acids or “heavy” media containing ^13^C_6_-lysine and ^13^C_6_,^15^N_4_-arginine [84]. Cells were then infected with VSV-pseudotype HIV (strain NL4-3) virus at a multiplicity of infection of 5. Both “light” and “heavy” cell populations were infected or mock infected in duplicate. Infected cells were either treated with MG-132 (Millipore) at 10 μM for 4 hours prior to harvest or were left untreated. Cells were harvested 48 hours after infection by lysis in a buffer containing 8 M urea, 0.1 M Tris pH 8.0, 100 mM NaCl, and protease inhibitors (Complete EDTA-free, Roche). 10 mg of protein as measured by a Bradford assay (Quick Start Bradford reagent, Bio-Rad) was subjected to trypsin digestion and ubiquitin remnant immunoaffinity purification with 250 μg of ubiquitin remnant antibody (Cell Signaling) as previously described [85]. The diglycine remnant against which the ubiquitin remnant antibody was raised is also exposed for NEDD8 and ISG15-modified proteins. 100 μg of each trypsin-digested Jurkat lysate was reserved for protein abundance analysis. Peptides were fractionated by hydrophilic interaction chromatography (HILIC). The peptides were injected onto a TSKgel amide-80 column (Tosoh Biosciences, 2.0 mm x 15 cm packed with 5 μm particles) equilibrated with 10% HILIC Buffer A (2% ACN, 0.1% TFA) and 90% HILIC Buffer B (98% ACN, 0.1% TFA) using an AKTA P10 purifier system. The samples were then separated by a one-hour gradient from 90% HILIC Buffer B to 55% HILIC Buffer B at a flow rate of 0.3 ml/min. Fractions were collected every 1.5 min and combined in 12 fractions based on the 280 nm absorbance chromatogram. Fraction were evaporated to dryness and reconstituted in 20 μl of 0.1% formic acid for mass spectrometry analysis.

For ubiquitination analysis in HEK293 cells, a HEK293 cell line was engineered to express HIV (strain NL4-3) with a tetracycline-responsive promoter element using the pTRE-Tight TetOn system (Clontech) [86] and a self-inactivating deletion in the 3’ UTR to prevent second round infection (publication forthcoming). All cells were treated with interferon alpha 1 (Cell Signaling) at 30 ng/ml and for +Dox samples HIV was induced by the addition of doxycycline (Sigma) at 1 μg/ml for 48 hours. Cells were either treated with MG-132 (Millipore) at 10 μM for 5 hours prior to harvest or were left untreated. All HEK293 experiments were performed in duplicate. 1 mg of protein as measured by a Bradford assay (Quick Start Bradford reagent, Bio-Rad) was subjected to trypsin digestion and ubiquitin remnant immunoaffinity purification with 31 μg of ubiquitin remnant antibody (Cell Signaling) as previously described [85].

All samples were analyzed on a Thermo Scientific LTQ Orbitrap Elite MS system equipped with an Easy nLC-1000 HPLC and autosampler system that is capable of maintaining backpressures of up to 10,000 psi for high-resolution chromatographic separations. The HPLC interfaces with the MS system via a nanoelectrospray source. Samples were injected onto a C18 reverse phase capillary column (75 um inner diameter x 25 cm length, packed with 1.9 um ReproSil Pur C18-AQ particles). Peptides were then separated by an organic gradient from 5% to 30% ACN in 0.1% formic acid over 112 minutes at a flow rate of 300 nL/min. The mass spectrometer collected data in a data-dependent fashion, collecting one full scan in the Orbitrap at 120,000 resolution followed by 20 collision-induced dissociation MS/MS scans in the dual linear ion trap for the 20 most intense peaks from the full scan. Dynamic exclusion was enabled for 30 seconds with a repeat count of 1. Charge state screening was employed to reject analysis of singly charged species or species for which a charge could not be assigned.

Raw mass spectrometry data were analyzed using the MaxQuant software package (version 1.3.0.5) [87]. Data were matched to the SwissProt human reference protein database (downloaded on May 15, 2013). MaxQuant was configured to generate and search against a reverse sequence database for false discovery rate calculations. Standard variable modifications were allowed for methionine oxidation, and protein N-terminus acetylation. When searching ubiquitination samples, a variable modification was also allowed for lysine ubiquitin remnant addition. A fixed modification was indicated for cysteine carbamidomethylation. Full trypsin specificity was required. The first search was performed with a mass accuracy of +/- 20 parts per million and the main search was performed with a mass accuracy of +/- 6 parts per million. A maximum of 5 modifications were allowed per peptide. A maximum of 2 missed cleavages were allowed. The maximum charge allowed was 7+. Individual peptide mass tolerances were allowed. For MS/MS matching, a mass tolerance of 0.5 Da was allowed and the top 6 peaks per 100 Da were analyzed. MS/MS matching was allowed for higher charge states, water and ammonia loss events. Data were searched against a concatenated database containing all sequences in both forward and reverse directions with reverse hits indicating the false discovery rate of identifications. The data were filtered to obtain a peptide, protein, and site-level false discovery rate of 0.01. The minimum peptide length was 7 amino acids. Results were matched between runs with a time window of 2 minutes for technical duplicates.

### Statistical analysis of proteomics data

The HIV ubiquitination proteomics results from the HEK293 and Jurkat cells were analyzed using an in-house computational pipeline built for the analysis of post-translationally modified peptides with mixed effect models, implemented in the MSstats (v2.3.4) Bioconductor package [88]. For both datasets, protein identifiers were converted into modification site identifiers, contaminant and false positive MaxQuant search results were removed, and all samples were normalized per cell line by median-centering the log2-transformed MS1-intensity distributions. Then, the MSstats groupComparison function was run on the Jurkat dataset with the following options: no interaction terms for missing values, no feature interference, unequal intensity feature variance, restricted technical and biological scope of replication. The HEK293 dataset was processed in a similar fashion with the following options: imputation of missing values with the mean minimal observed MS1-intensity across samples, no feature interference, unequal intensity feature variance, restricted technical and biological scope of replication. We opted not to impute missing values for the SILAC Jurkat dataset since they are less prevalent due to the nature of SILAC assays.

### Structural bioinformatics

In addition to the ubiquitination proteomics data that we collected in Jurkat and HEK293 cells, we used the PTMfunc database of protein PTMs [60] to identify other PTMs present on human protein kinases. Functional annotations for some of these PTMs are included in the PTMfunc database [60]. In-house scripts were used to map PTMs from all human kinases onto the PKA and ZAP-70 structures based on an alignment using the ClustalW2 multiple sequence alignment software from EMBL-EBI [89–91]. When mapping PTMs onto the PKA or ZAP-70 structures, any PTM in a location with no alignment to the mapping protein was omitted.

To determine whether a ubiquitination site is associated with proteasomal degradation, we used data collected in the presence of a proteasome inhibitor (MG-132). Sites whose abundance was at least two-fold greater in the presence of a proteasome inhibitor compared to control were categorized as proteasome sensitive, whereas those with a difference of less than two-fold were categorized as proteasome insensitive. If the same site was proteasome sensitive in one cell line or condition and proteasome insensitive in another, we categorized it as neither. For sites that were significantly more abundant in the presence of HIV, we determined the proteasome sensitivity based on MG-132 sensitivity in the presence of HIV only, since the level of ubiquitination without HIV present might be very low. We determined whether differences between groups (such as proteasome-sensitive and insensitive ubiquitin modifications) are significant by using the function *prop*.*test* in R [92] to find a p-value, which tests whether the proportions in different group are the same.

### Molecular dynamics simulations

We performed all-atom MD simulations starting from 9 different ZAP-70 kinase domain constructs. We had five constructs built from the active-state crystal structure: no modification (control), monoubiquitinated at K377, monoubiquitinated at K476, acetylated at K377, and with an immunoglobulin (Ig) domain from Cardiac Myosin Binding Protein C attached at K377. We also built four constructs from the inactive-state crystal structure: control, monoubiquitinated at K377, monoubiquitinated at K476, and acetylated at K476.

#### Model building

The starting structure for the active ZAP-70 kinase domain was the crystal structure 1U59 [81]. We replaced the C-terminal residues 601–614 with those from the inactive structure 4K2R [77] because the 1U59 construct has a His-tag at the C-terminus. We used the PLOP homology modeling software [93] to predict the conformation of residues 359–361, which are missing in the 1U59 structure. We removed the crystallographic waters and the staurosporine ligand and then used Maestro [94] to predict the protonation states of the histidine side chains. For the inactive ZAP-70 kinase domain we used the crystal structure of the autoinhibited protein, 4K2R [77]. We removed residues 1–327, the SH2 domains, from this structure leaving only the kinase domain. We replaced the missing activation loop, residues 478–518, using the active ZAP-70 structure (1U59). Side chains with steric clashes were adjusted in Maestro. We removed the phosphoaminophosphonic acid-adenylate ester molecule, phosphate ion, and magnesium ion from the crystal structure to make the inactive system as similar as possible to the active kinase system.

We prepared the ubiquitinated ZAP-70 structures using a crystal structure of diubiquitin, 3NS8 [95]. In this structure, the C-terminus of the chain A ubiquitin is covalently attached to the K48 side chain of the chain B ubiquitin. We aligned K48 of 3NS8 chain B with the lysine residue we wanted to ubiquitinate on the ZAP-70 structure. We then manually replaced the ZAP-70 lysine coordinates with those from the aligned 3NS8 chain B K48 residue in the PDB file and changed this residue’s name to UBK. We added the aligned 3NS8 chain A coordinates to the ZAP-70 PDB file. To create parameters for the UBK modified residue, we started with the parameters for acetylated lysine [96], which has a chemical structure and partial charges similar to ubiquitinated lysine. The parameter file was manually modified to contain the correct number of atoms for ubiquitinated lysine, and the NZ and HZ atomic charges were decreased slightly, to -0.4957 and 0.3082 respectively, to make the entire residue neutral. We used the *LEaP* module from Amber [97] to create library files for the UBK residue (S1 File).

We prepared the acetylated ZAP-70 structures by modifying the PDB file manually to change the residue name from LYS to ACK. We used previously published parameters to simulate the acetylated lysine in Amber [96]. We used the *LEaP* module from Amber [97] to create library files for the ACK residue (SI). To attach the Ig domain at K377 we used the 3CX2 PDB structure of the Myosin Binding Protein C Ig domain [98]. We aligned its C-terminal residues (257–258) with the C-terminal structured residues of ubiquitin (72–73) on the ZAP-70 K377-ubiquitinated structure and rotated the Ig domain to relieve steric clashes with the ZAP-70 kinase. We then manually replaced the ubiquitin coordinates in the ubiquitinated structure with the Ig domain coordinates, keeping the C-terminal residues of ubiquitin that make up the linker (74–76) but changing R74 to a glycine. We used the same parameters for the UBK residue that we had for the ubiquitinated constructs.

#### Simulation protocol

*LEaP* was used to prepare all structures for simulation, surrounding the protein with 10 Å of water on all sides and adding Cl^-^ ions to neutralize the system. The number of water molecules added varied depending on the construct (S1 Table). The structure was energy minimized and then equilibrated at constant volume while raising the temperature to 300 K. A constant pressure simulation was then run for 1 ns to equilibrate the system density. Dimensions of the periodic box after density equilibration for each construct are given in S1 Table. All MD simulations were performed using the *pmemd* module in the Amber package [97], Amber99SB force field [99], and explicit TIP3P water representation [100]. The Andersen thermostat was used to hold the temperature constant at 300 K [101,102], and the Berendsen barostat with isotropic position scaling and a relaxation time of 1 ps to hold the system at 1 bar [103]. Particle-mesh Ewald procedure was used to handle long-range electrostatic interactions with a non-bonded cutoff of 9 Å. Simulations were run on GPUs, which gave the most efficient performance. After the initial 1 ns equilibration, 8 independent 10-ns simulations were carried out starting from the equilibrated structure, but with new initial, randomized, velocities. These 10 ns simulations were considered additional equilibration. The final structures from each of these simulations were used to start 4 new independent simulations of 10 ns, resulting in a total of 32 independent 10-ns simulations. The trajectories, spanning a total of 320 ns of simulation, were analyzed for each system with snapshots taken every picosecond. In addition we ran a single 100 ns simulation for each of the unmodified ZAP-70 constructs (starting from the active state crystal structure and starting from the inactive state crystal structure). We also ran a single 560 ns simulation for the active state ZAP-70 construct ubiquitinated at K377 and for the inactive state ZAP-70 construct ubiquitinated at K476. These longer simulations were also analyzed by taking snapshots every picosecond.

#### Molecular dynamics analysis

We analyzed the MD trajectories using the *cpptraj* module of Amber [97]. The secondary structure of the ZAP-70 C-helix was determined using the DSSP criteria described by Kabsch and Sander [104]. In-house scripts were used to analyze the MD trajectories and the R statistical computing and graphics language [92] was used to plot the structural fluctuations of the ZAP-70 kinase domain. All histograms were computed by combining all independent trajectories for a given construct (snapshots spaced every picosecond) and binning over this total ensemble of structures. To determine whether the distribution of various structural metrics differed between simulations of different ZAP-70 constructs, we treated the mean value from each of the 32 independent simulations as a single data point. We then used the function *wilcox*.*test* in R, which performs a Wilcoxon rank sum test, to test the difference between the distributions for different constructs. We performed the statistical test on the mean of each independent simulation because the structures within each 10-ns simulation are correlated. We chose the Wilcoxon rank sum test because it is valid for data that are not normally distributed.

Ending conformations from the ubiquitinated ZAP-70 simulations, shown in S1 Fig, were selected by choosing from among the last conformations sampled in the 32 production simulations. Specifically, we choose the structure (among those 32 end-points) that differed the most from the starting ZAP-70 crystal structure based on the F349Cα-D379Cβ distance, which indicates the position of the C-helix. For the active K377-ubiquitinated simulations the ending structure with the longest distance (14.9 Å) was chosen, and for the inactive K476-ubiquitinated simulations the ending structure with the shortest distance (7.6 Å) was chosen. To create contact maps, we used ptraj and python scripts to calculate the percent of the snapshots from our simulations (taken every 100 ps) where each pair of residues was in contact. We plotted this matrix in R and annotated it with the values for the most populated contacts. We define residue contacts as a separation of less than 10 Å averaged over the residue. Solvent accessible surface area of the kinase was calculated using the *surf* command in *cpptraj*, which uses the LCPO method [105]. All protein structure figures were created using VMD [106].

## Results

### Kinase ubiquitination

**Fig 1:**
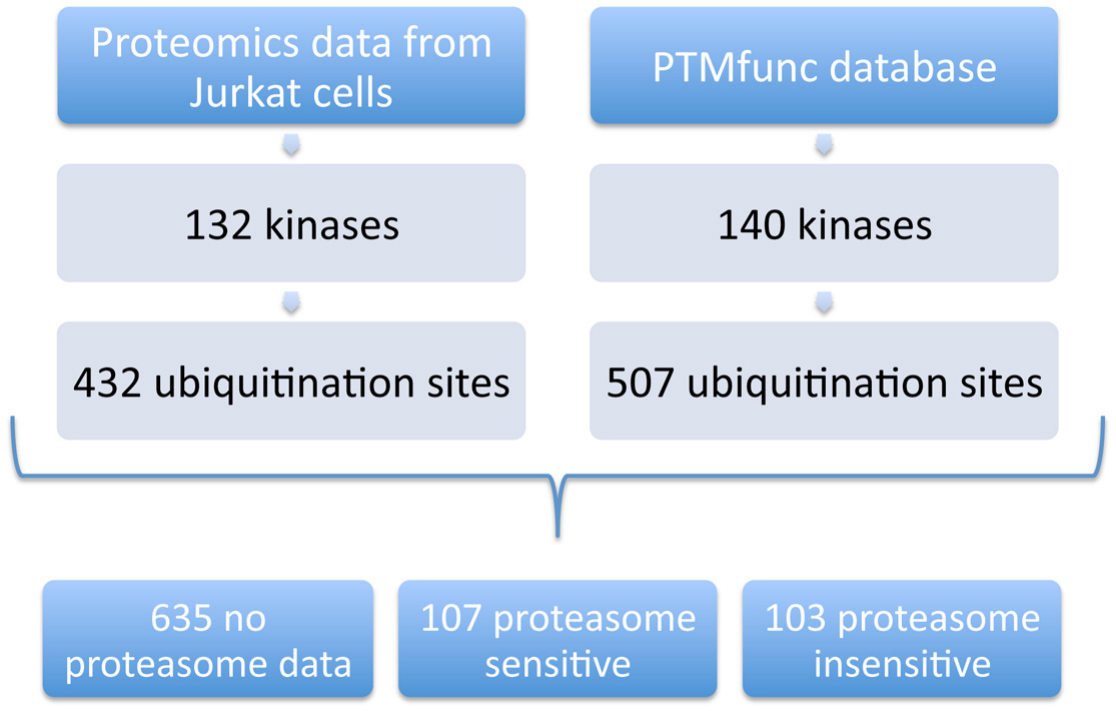
Flowchart of proteomics data analysis.

As part of a larger study of HIV-host interactions, data were generated to investigate changes in ubiquitination in response to HIV infection and proteasome inhibition (manuscript in preparation). Ubiquitination sites (indistinguishable from NEDD8 or ISG15 sites) were identified using ubiquitin remnant immunoaffinity enrichment and quantitative mass spectrometry in Jurkat and HEK293 cells, both in the presence and absence of HIV infection [107]. The HEK293 cells were treated with interferon, to activate the expression of known antiviral substrates of ubiquitination and proteasomal degradation, while the Jurkat cells were not. Indeed, ubiquitination of APOBEC3C and the cellular receptor CD4 (stably transfected in these cells) were detected in HEK293 experiments responding to the expression of HIV and only in the presence of a proteasome inhibitor (S2 Fig). As interferon treatment also induces expression of the ubiquitin-like protein ISG15, which generates an indistinguishable ubiquitin remnant epitope, we used the data from the Jurkat cells, to examine ubiquitination on kinases, including sensitivity to proteasome inhibition. The Jurkat cells were not interferon treated and thus the sites identified reflect ubiquitination sites that are not confounded by the possibility of being ISG15 modifications. This is true even in the HIV infected Jurkat cells since HIV prevents the activation of interferon stimulated genes by suppressing IRF3 activity [108]. This was verified by checking the abundance of ISG15 and proteins known to be induced by interferon, none of which increased significantly with HIV infection (S1 Dataset). A total of 432 sites were identified on 132 protein kinases in the Jurkat cell studies (Fig 1). Combining this proteomics data with those in the PTMfunc database [60], we found that 201 human protein kinases are ubiquitinated at one or more sites, with a total of 845 ubiquitination sites; 107 of these ubiquitin modifications are proteasome sensitive, and 103 are proteasome insensitive (the proteasome sensitivity of the remaining sites is unknown). All 103 of the proteasome insensitive ubiquitination sites were ubiquitinated even in the absence of HIV infection, indicating that they do not result from nonspecific ubiquitination caused by infection. The frequency of HIV dependence for ubiquitination sites is reported in supporting information (S2 Table). The 103 proteasome insensitive ubiquitination sites are unlikely to be associated with degradation by the proteasome, and may serve another function such as regulation of kinase activity. A full list of all ubiquitination sites is provided in the supporting information (S2 Dataset).

We found that most ubiquitin modifications occur on a folded domain (Table 1), in contrast to phosphorylation sites, which are much more likely to be located in unstructured regions (Table 1). This difference is statistically significant (p-value < 2.2x10^-16^ with *prop*.*test*) and has been observed in studies of global ubiquitination [60,62–64], but the underlying reason is not clear. One possible explanation is that the E3 ligase requires a structured domain for ubiquitin attachment, whereas kinases require the segment around the phosphorylation site to be in an extended conformation. Many of the phosphorylation sites located in unstructured regions are not believed to be functional [60,109,110]. Since most ubiquitin modifications are attached to a structured region of the kinase, we speculate that those that are proteasome insensitive may regulate the kinase by affecting the stability, flexibility, or conformation of that structured region.

**Table 1:** Comparison of proteasome-sensitive and proteasome-insensitive ubiquitination and phosphorylation sites across human protein kinases. We have separated out the data from HEK293 cells because they may also include some ISG15 modifications.

**Jurkat + PTMfunc**	**On kinase domain**	**On any folded domain**	**Residue conserved**	**Modification conserved**	**Near phos. site**	**Near phos. hot spot**
**% of proteasome-sensitive ubiquitination sites**	52%	78%	87%	55%	11%	7%
**% of proteasome-insensitive ubiquitination sites**	42%	79%	81%	50%	17%	5%
**% of all ubiquitination sites**	47%	72%	80%	48%	11%	3%
**% of phosphorylation sites**	17%	28%	59%	44%	NA	NA
**HEK293**	**On kinase domain**	**On any folded domain**	**Residue conserved**	**Modification conserved**	**Near phos. site**	**Near phos. hot spot**
**% of proteasome-sensitive ubiquitination sites**	*56%	*80%	79%	44%	12%	4%
**% of proteasome-insensitive ubiquitination sites**	*36%	*60%	79%	33%	15%	0%
**% of all ubiquitination sites**	46%	68%	79%	39%	12%	2%

* indicates a significant difference between the proteasome-sensitive and proteasome-insensitive percentages using the function *prop*.*test* in R. A conserved residue is defined as occurring more than 40 times out of 832 kinases, and a conserved modification is defined as occurring more than 5 times. “Near” is defined as within four residues of the ubiquitinated residue. Phosphorylation hot spots are those sites where phosphorylation is believed to play a role in function, as determined by the PTMfunc database. We also have data on the effect of HIV expression on ubiquitination, reported in the supporting information (S2 Table).

**Fig 2:**
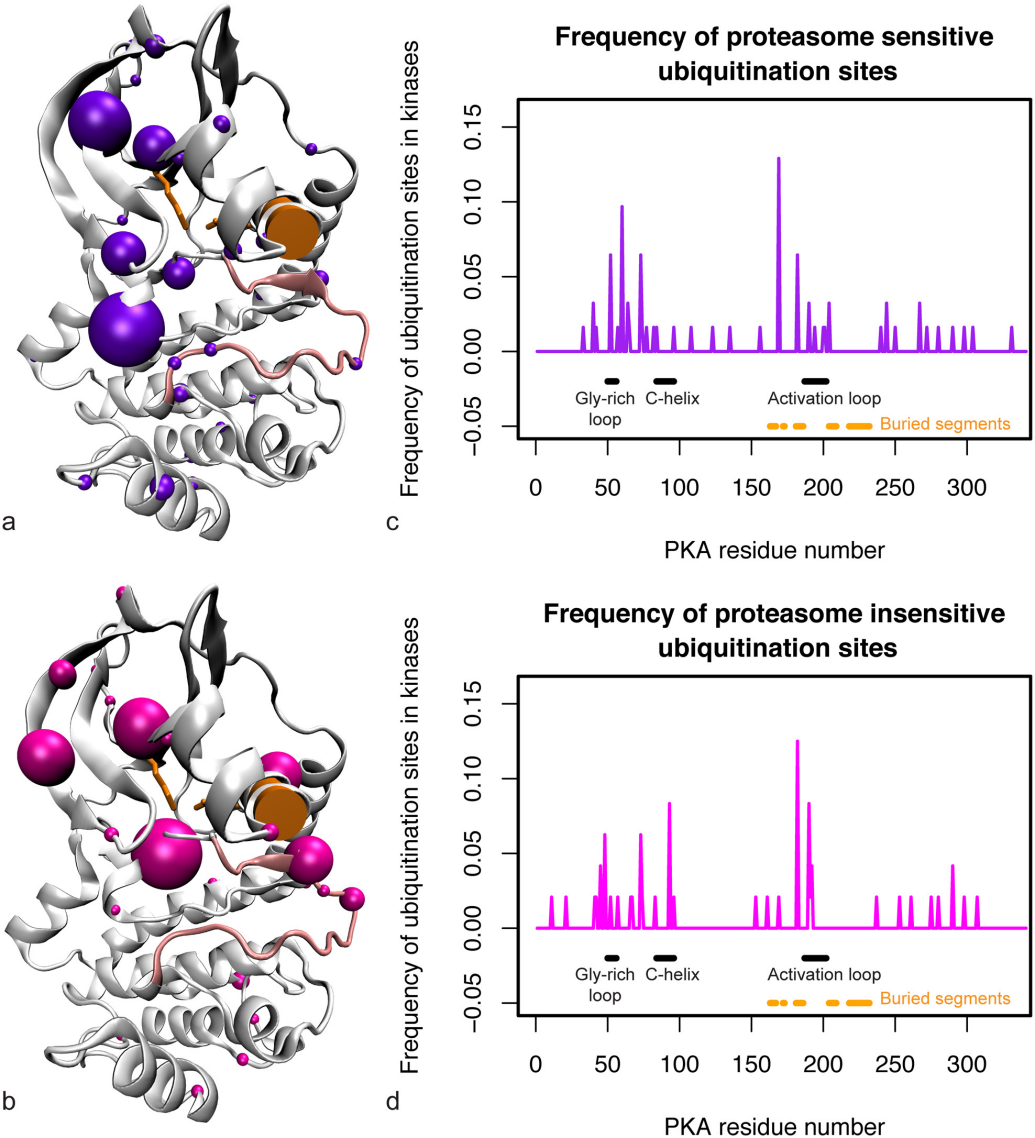
All proteasome-sensitive and proteasome-insensitive sites from human kinases. (a) Proteasome-sensitive sites (purple spheres) mapped onto the PKA structure. Sphere size is proportional to the fraction of ubiquitin modifications that occur at that site. (b) Proteasome-insensitive sites (pink spheres) mapped onto the PKA structure. Sphere size is proportional to the fraction of ubiquitin modifications that occur at that site. The C-helix is highlighted by an orange cylinder, K369 on strand β3 of the N-lobe and E386 on the C-helix (forming the salt bridge that holds the C-helix in place) are shown with orange sticks, and the activation loop is shown in light pink. (c) Frequency of proteasome-sensitive ubiquitination sites on the kinase domain. (d) Frequency of proteasome-insensitive ubiquitination sites on the kinase domain. The ubiquitination sites across all kinases are mapped onto the PKA sequence on the x-axis, normalized by the total density on the kinase domain.

Fig 2 maps all of the proteasome-sensitive and proteasome-insensitive ubiquitin modifications observed in kinase domains onto the protein kinase A (PKA) structure. Ubiquitin modifications occur on essentially all solvent exposed regions of the kinase domain, but are enriched on the region of the N-lobe opposite the C-helix and near the glycine loop, at the N-terminal end of the activation loop, and on the region of the C-lobe distal to the substrate pocket (Fig 2). These data are consistent with another study that found the glycine-rich loop and the region N-terminal to the activation loop of kinases to be enriched in ubiquitination sites [61]. Both proteasome-sensitive and proteasome-insensitive modifications are concentrated in these functionally important regions, but certain residues are more commonly associated with proteasome-insensitive ubiquitination. In particular there are residues associated with proteasome-insensitive ubiquitination on the N-terminal side of the glycine-rich loop, on the N-terminal side of the activation loop (near the DFG motif), and one on the C-helix itself. Ubiquitination at these sites may cause specific conformational changes that affect kinase activity or interaction with other proteins.

We also examined whether kinase ubiquitination sites are conserved across different human kinases. We found that about half of modification sites are common across more than 5 of the 206 ubiquitinated kinases, and that the ubiquitinated residue is usually conserved as a lysine across more than 40 other human kinases (Table 1). As a point of comparison (null hypothesis), if the ubiquitination sites were chosen randomly from all the lysine residues on all the kinases, the percentage occurring at a conserved residue would be 29%. Instead, we observe that 80% of ubiquitin modifications occur at a conserved residue, indicating that these residues are preferred. Wagner *et al*. also reported that ubiquitinated residues are more conserved than other lysines, possibly because they tend to be in structured regions [62]. We hypothesized that proteasome-insensitive sites would be more conserved than those affected by proteasome degradation; however, we did not find any significant differences between their conservation. This implies that, whether or not ubiquitination is associated with proteasomal degradation, the ubiquitin modification tends preferentially to occur at conserved sites, many of which are critical to kinase structure or function.

We also tested whether other PTMs, such as phosphorylation, co-localize with ubiquitination sites, and determined that 11% of ubiquitination sites occurred within 4 residues of a phosphorylation site on the same protein (Table 1). This fraction is higher than the 3% of collocated sites that would be found if ubiquitination sites were chosen randomly from among all lysine residues. Similarly, 3% of ubiquitination sites are located near a residue identified as a functional phosphorylation hot spot (sites where phosphorylation is believed to play a role in function, as determined by the PTMfunc database) [60], rather than the 0.3% expected from a random distribution (Table 1). No difference was observed between proteasome-sensitive and proteasome-insensitive ubiquitination sites with regard to whether they are located near a phosphorylation site or hot spot.

**Fig 3:**
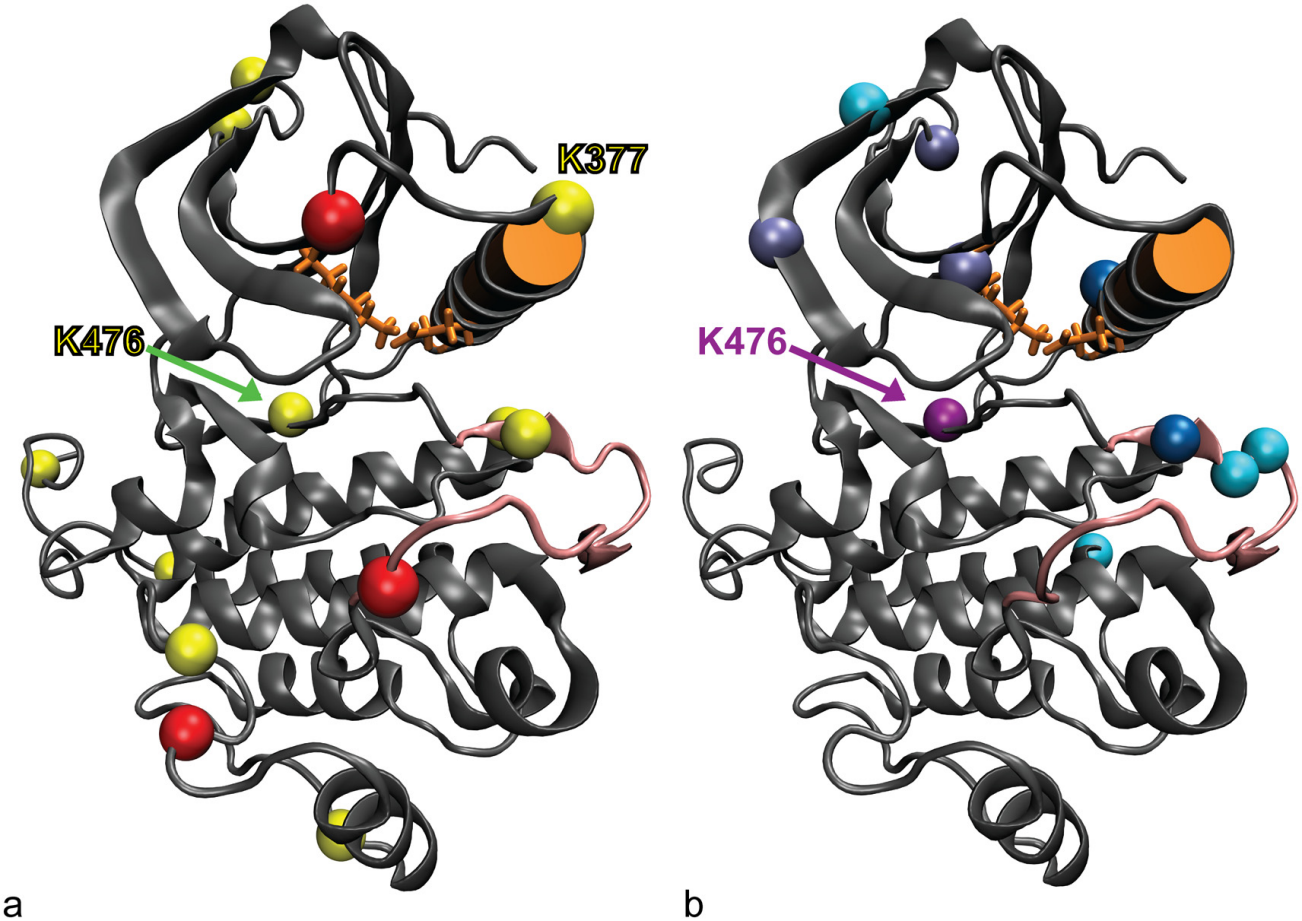
ZAP-70 ubiquitination. (a) ZAP-70 kinase domain ubiquitination sites. Proteasome-insensitive sites are shown in yellow, proteasome-sensitive sites and those where proteasome sensitivity is unknown are shown in red. K377 and K476 are labeled. (b) Most common proteasome-insensitive kinase ubiquitination sites mapped onto the ZAP-70 kinase structure. The color indicates how many kinases are ubiquitinated at this site (light blue = 2, grey blue = 3, dark blue = 4, mauve = 6). Not all of the sites are ubiquitinated on ZAP-70, but the most common site, K476, is. In both figures the C-helix is represented with an orange cylinder, K369 on strand β3 of the N-lobe and E386 on the C-helix (forming the salt bridge that holds the C-helix in place) are shown with orange sticks, and the activation loop is shown in pink.

The kinase with the largest number of proteasome-insensitive ubiquitination sites is ZAP-70 (Table 2). For this reason, and because its structure, function and regulation are well studied, we chose ZAP-70 for an initial study of how proteasome-insensitive ubiquitination might affect kinase structure and dynamics. Fig 3 shows the locations of both proteasome-sensitive and proteasome-insensitive ubiquitination sites on ZAP-70. The first site we chose for modeling ZAP-70 ubiquitination was K377 (Fig 3), a site close to the C-helix, because the C-helix position is crucial for kinase activity. We hypothesized that a ubiquitin moiety attached near the C-helix might disrupt its position and shift the kinase conformation away from the active structure. To choose a second ZAP-70 ubiquitination site for modeling, we examined the proteasome-insensitive ubiquitination frequency of the various sites across human kinases (Fig 3). The most common site for proteasome-insensitive ubiquitination is K476 (ZAP-70 numbering), which is ubiquitinated on 6 kinases: ZAP-70, Lck, MAPK9, MAPK8, MAPK10, and CDK5. The K476 ubiquitination site is not as close to the C-helix and active site as K377, and therefore we hypothesized that simulations of K476-ubiquitinated ZAP-70 would have a smaller effect on active site conformation and dynamics, but might still disrupt the active state allosterically. Both K377 and K476 were ubiquitinated on ZAP-70 in cells not infected with HIV, so these modifications are likely relevant to normal ZAP-70 kinase regulation.

**Table 2:** Kinases with the most proteasome-insensitive and proteasome-sensitive ubiquitination sites.

Protein	Uniprot ID	Proteasome-insensitive sites	Proteasome-sensitive sites	Sites without proteasome data
Kinases with the most proteasome-insensitive ubiquitination sites				
Tyrosine-protein kinase ZAP-70	P43403	12	1	6
Tyrosine-protein kinase LCK	P06239	7	1	5
Eukaryotic elongation factor 2 kinase	O00418	5	0	1
Serine/threonine-protein kinase PAK 2	Q13177	5	0	2
Serine/threonine-protein kinase PAK 3	O75914	4	0	0
Protein kinase C alpha type	P17252	3	0	5
Glycogen synthase kinase-3 alpha	P49840	3	0	0
Protein kinase C delta type	Q05655	3	1	3
Kinases with the most proteasome-sensitive ubiquitination sites				
Cyclin-dependent kinase 1	P06493	0	11	8
Aurora kinase B	Q96GD4	0	6	8
Cyclin-dependent kinase 2	P24941	0	6	8
Cyclin-dependent kinase 6	Q00534	1	5	7
Tyrosine-protein kinase ITK/TSK	Q08881	0	5	11
Beta-adrenergic receptor kinase 1	P25098	0	4	6
Dual specificity mitogen-activated protein kinase kinase 2	P36507	0	3	8
Maternal embryonic leucine zipper kinase	Q14680	1	3	11
Serine/threonine-protein kinase TBK1	Q9UHD2	0	3	5
Integrin-linked protein kinase	Q13418	2	3	6

### ZAP-70 MD simulations

**Fig 4:**
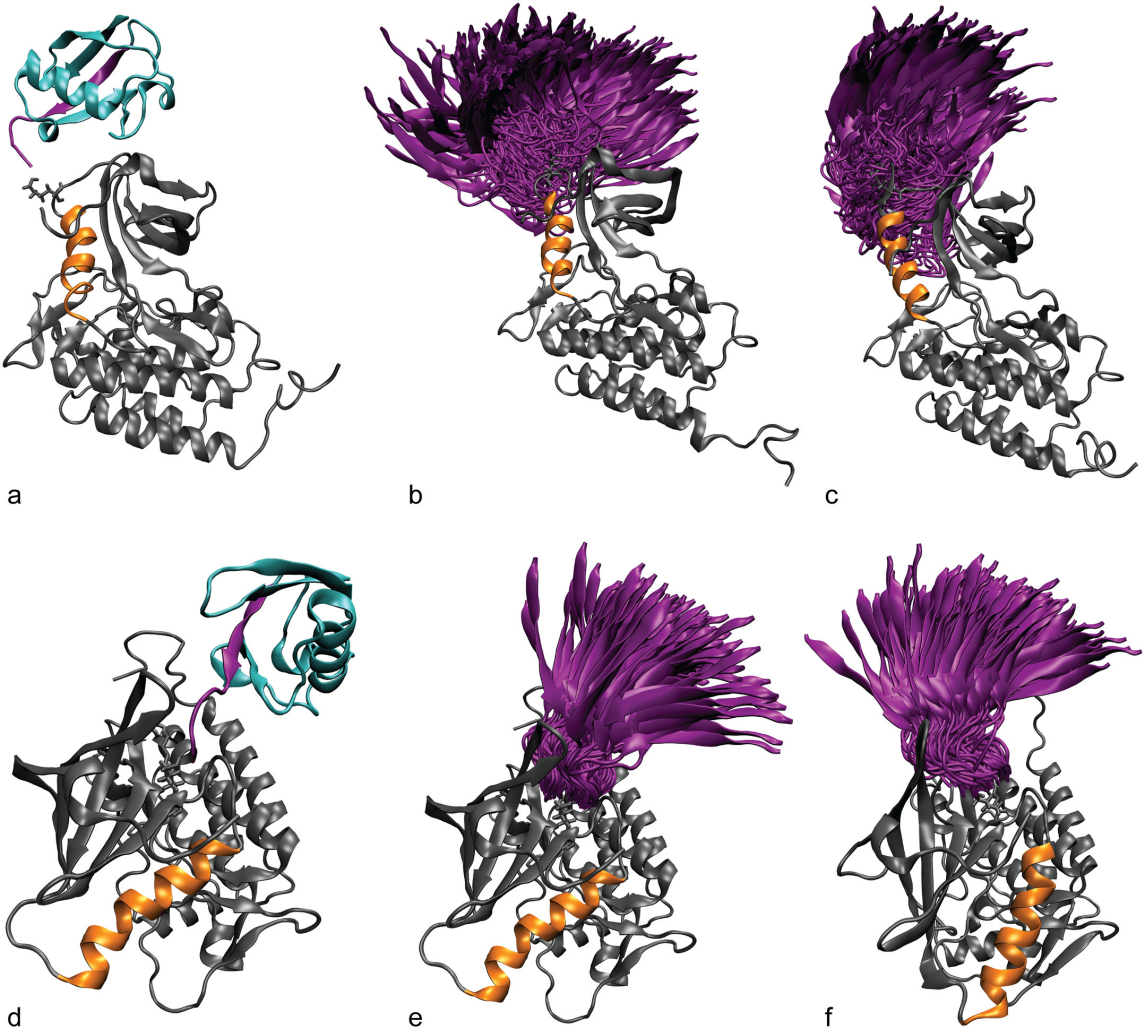
Ubiquitin attachment and sampling. The starting structure for simulations of the active ZAP-70 kinase ubiquitinated at K377 (a) and K476 (d). The C-helix is shown in orange and the ubiquitin molecule is shown in cyan. The C-terminal β-strand of ubiquitin is highlighted in purple. The diversity of orientations that the ubiquitin moiety samples, starting from the K377-ubiqutiinated active (b) and inactive (c) structures and K476-ubiquitinated active (e) and inactive (f) structures is summarized by showing only the orientation of the C-terminal β-strand (purple) relative to the kinase, for snapshots separated by 1 ns.

We conducted simulations of ZAP-70 starting from the active and inactive-state crystal structures with a ubiquitin attached at K377 or K476. K377 is near the N-terminal end of the C-helix (Fig 4(a)), and K476 lies on the opposite side of the N-lobe from the C-helix, near the hinge region (Fig 4(d)). In all simulations of ubiquitinated ZAP-70, we attached only a single ubiquitin molecule, as this is the minimal possible modification. The proteomics data does not determine the number of ubiquitin molecules or, in the case of polyubiquitination, the attachment residue. Since these modifications were proteasome insensitive, they are most likely to be K63-linked or monoubiquitinated. We acknowledge that K63-linked polyubiquitination may have different effects from monoubiquitination, but we anticipate that there may be commonalities, especially since K63-linked polyubiquitin chains are extended, leaving only the proximal ubiquitin domain to interact with the target protein. As a practical matter, simulating a polyubiquitin attachment would also greatly increase the computational expense. For all ZAP-70 simulations we used a construct that was not phosphorylated at Y493 on the activation loop. The available crystal structures did not include phosphorylation at Y493, and we did not want our simulations to be complicated by the effects of two modifications away from the crystal structure. Also, although ZAP-70 must be phosphorylated at Y493 to be fully active, there is no data to indicate whether ZAP-70 ubiquitination occurs prior to or following phosphorylation, so a ubiquitinated but unphosphorylated ZAP-70 may be relevant *in vivo*.

In our simulations, the ubiquitin domain interacted only transiently with the ZAP-70 kinase domain and sampled many different relative orientations (Fig 4), demonstrating that the starting position of the ubiquitin molecule was not important. The range of orientations sampled by ubiquitin attached at K476 is more restricted than those sampled by ubiquitin attached at K377, probably because K476 is surrounded by other parts of the kinase structure that interfere sterically with the ubiquitin motion.

#### K377 and K476 ubiquitination have opposite effects on the kinase conformation

We performed molecular dynamics on the ZAP-70 kinase domain ubiquitinated at either K377 or K476, as well as the unmodified control. For each ubiquitination site we performed simulations starting from the ZAP-70 “active” structure, and starting from the “inactive” structure (see Methods) for a total of 320 ns of simulations for each construct.

**Fig 5:**
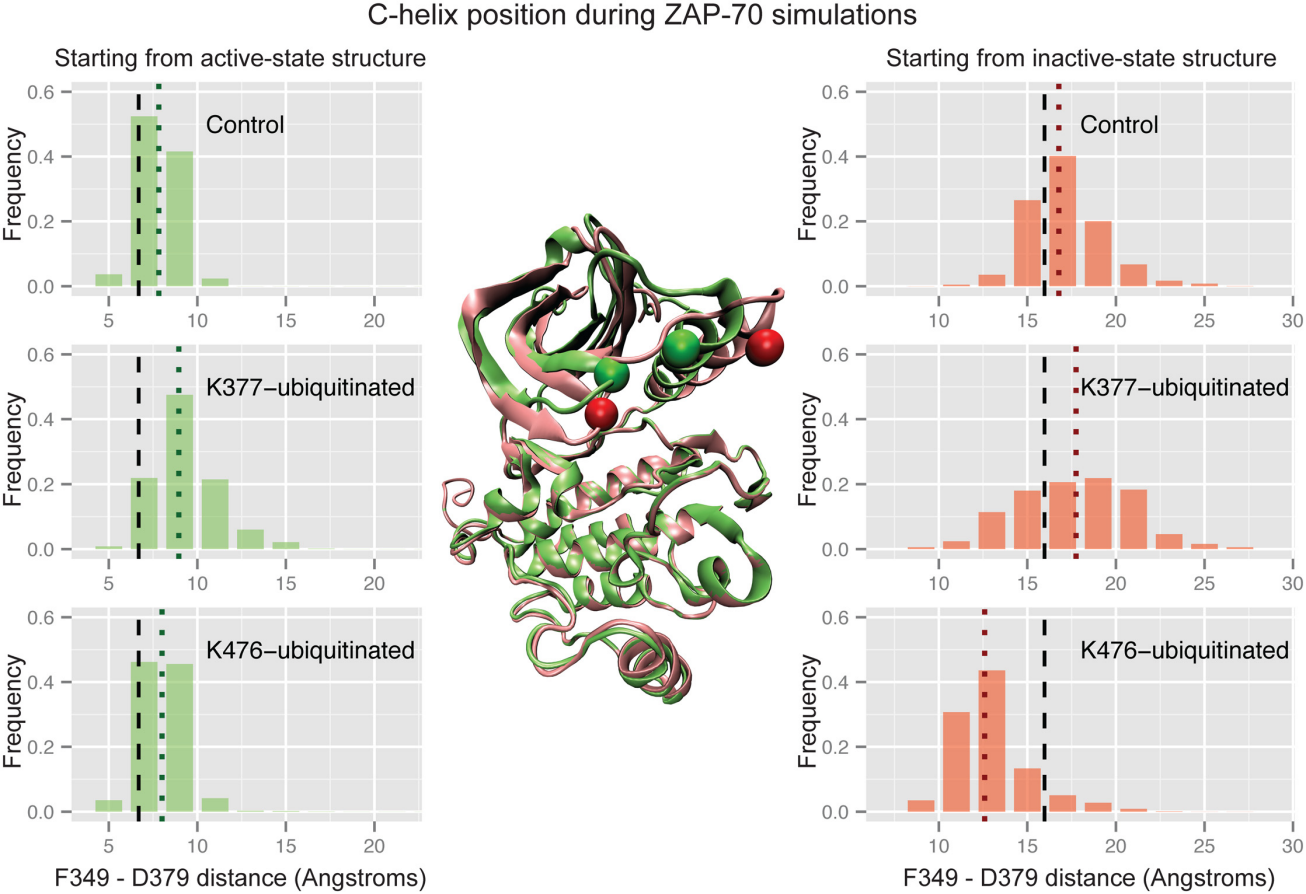
Ubiquitination affects ZAP-70 C-helix position. Histograms of the distance between the F349 Cα atom and the D379 Cβ atom during three sets of ZAP-70 simulations; control simulations, K377-monoubiquitinated, and K476-monoubiquitinated, both started from the active state structure (left) and the inactive, autoinhibited structure (right). The vertical dashed black lines indicate the distance observed in the starting crystal structure, while the dotted colored lines indicate the median value for each histogram. The histogram is created from combining all structures sampled in each of the 32 independent simulations with snapshots every picosecond. In the center, residues F349 and D379 are shown as spheres on the overlaid ZAP-70 active (green) and inactive (red) crystal structures.

The most striking differences between the ubiquitinated and control simulations of ZAP-70 occurred at the C-helix, which is critical for kinase activity. In the active structure, a salt bridge between K369 on strand β3 of the N-lobe and E386 on the C-helix holds the C-helix in place, while in the inactive structure its N-terminal end swings out from the rest of the N-lobe and rotates [75,76]. We measured this difference using a distance between a backbone atom on the glycine-rich loop (F349) and the Cβ atom of D379 on the N-terminal end of the C-helix to indicate how close the C-helix is to the rest of the N-lobe and how much it is rotated (Fig 5). In the control simulations starting from the active structure, the C-helix remains close to the N-lobe. Ubiquitination of the active-state structure at K377 (but not at K476) causes the C-helix to move away from the rest of the N-lobe, disrupting the conformation of the C-helix necessary for kinase activity (Fig 5). The difference in mean C-helix position between the control active and K377-ubiquitinated active simulations is significant according to the Wilcoxon rank sum test, with a p-value of 1.4x10^-10^ (all p-values comparing modified construct simulations to the control simulation are given in the supporting information S3 and S4 Tables). In the inactive control simulations the C-helix remains farther away from the rest of the kinase structure, and this distance is even slightly increased with ubiquitination at K377 (Fig 5). However, when the inactive structure is ubiquitinated at K476, the C-helix moves closer to the N-lobe (Wilcoxon rank sum test p-value = 3.1x10^-14^ compared to inactive control), approaching a position similar to that in the active state (Fig 5). Our simulations indicate that ubiquitination at two distinct sites on the ZAP-70 kinase domain result in opposite effects on kinase conformation and possibly activity.

**Fig 6:**
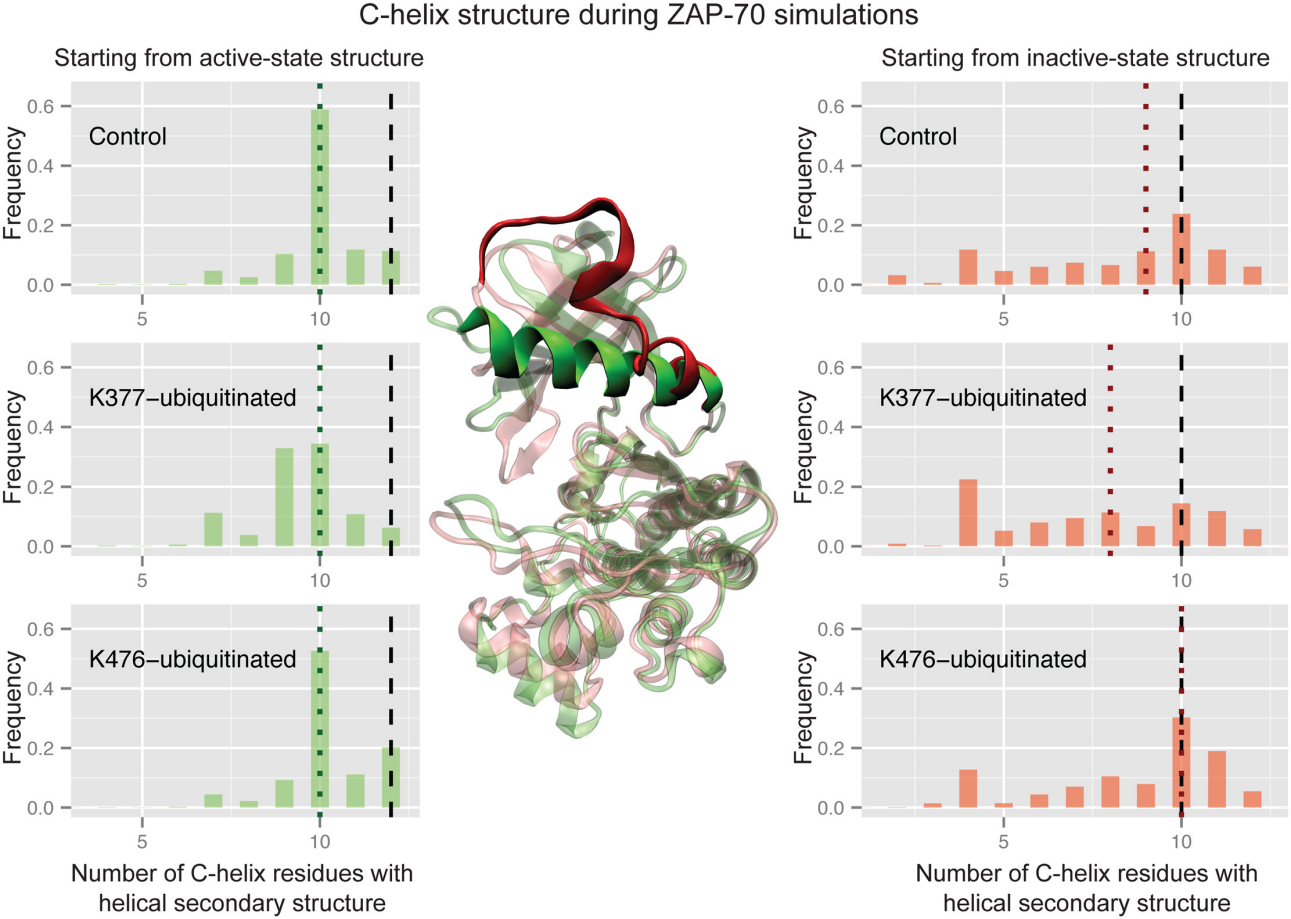
Ubiquitination affects ZAP-70 C-helix order. Histograms of the number of residues in the C-helix (380–393) that retain the helical conformation, using the DSSP definition, during three sets of ZAP-70 simulations; control simulations, K377-monoubiquitinated, and K476-monoubiquitinated, both started from the active state structure (left) and the inactive, autoinhibited structure (right). The vertical dashed black lines indicate the number of helical residues observed in the starting crystal structure, while the dotted colored lines indicate the median value for each histogram. The histogram is created from combining all structures sampled in each of the 32 independent simulations with snapshots every picosecond. In the center, the C-helix is highlighted on the ZAP-70 active crystal structure (green) and an overlaid structure from the inactive conformation simulations (red) where the C-helix is mostly unraveled (only 6 residues have helical secondary structure).

Ubiquitination of the active ZAP-70 at K377 also causes a significant difference in the secondary structure of the C-helix (Wilcoxon rank sum test p-value of 2.2x10^-6^ compared to the active control), which frequently becomes partially disordered, primarily at the N-terminal side (Fig 6). This was unexpected, because the C-helix shows the same secondary structure in both the active and inactive crystal structures. The unmodified ZAP-70 C-helix maintains at least 10 residues in a structured conformation in more than 80% of the simulated ensemble, as does the K476-ubiquitinated C-helix starting from the active state. Only ubiquitination at K377 leads to a significant decrease in C-helix structure from the active structure, with just 51% of the simulated ensemble maintaining 10 or more helical residues. In the control simulations starting from the inactive state, however, the highly solvent exposed C-helix partially and transiently unfolds, sometimes retaining only 2 residues in a helical secondary structure (Fig 6). Only 42% of the inactive-state control ensemble maintains 10 or more helical residues. The K377-ubiquitnated inactive-state simulations resulted in ensembles with an even more disordered C-helix (only 32% with 10 or more residues ordered). However, K476-ubiquitination of the inactive-state ZAP-70 resulted in a slight shift toward more ordered C-helix conformations, with 55% of the ensemble maintaining at least 10 helical residues. These differences are not statistically significant, but nonetheless are consistent with the trend of K476-ubiquitination shifting the ZAP-70 inactive-state C-helix more toward the active state. Interestingly, these changes in C-helix structure all occur without breaking or forming the salt bridge between the C-helix and N-lobe.

**Fig 7:**
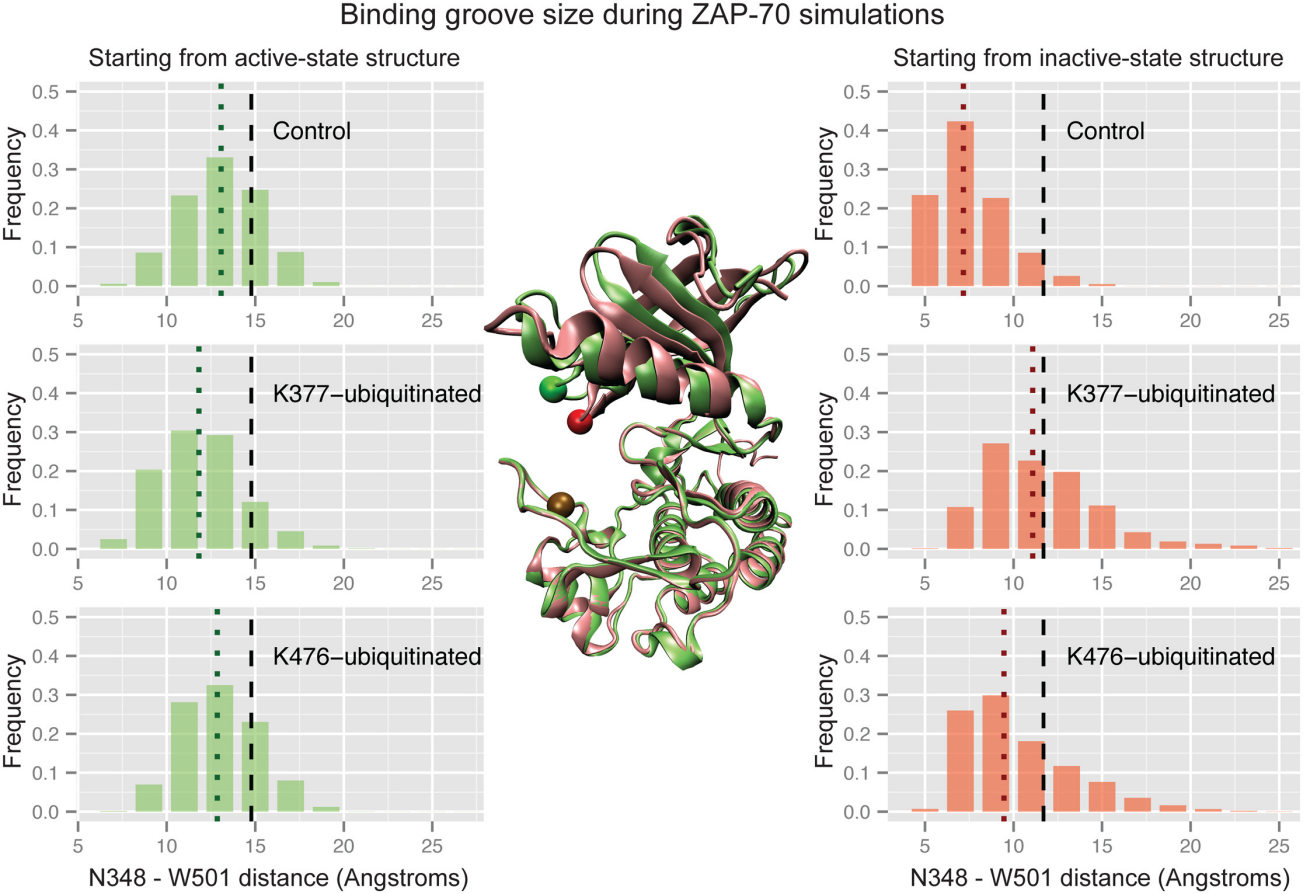
Ubiquitination affects ZAP-70 groove closure. Histograms of the distance between the N348 Cα atom and the W501 Cα atom during three sets of ZAP-70 simulations; control simulations, K377-monoubiquitinated, and K476-monoubiquitinated, both started from the active state structure (left) and the inactive, autoinhibited structure (right). The vertical dashed black lines indicate the distance observed in the starting crystal structure, while the dotted colored lines indicate the median value for each histogram. The histogram is created from combining all structures sampled in each of the 32 independent simulations with snapshots every picosecond. In the center, residues N348 and W501 are shown as spheres on the overlaid ZAP-70 active (green) and inactive (red) crystal structures.

It has also been noted that, in the inactive structure, the ZAP-70 N-lobe closes over the C-lobe, shutting the active site [75,76]. To quantify this conformational change we measure the distance between the backbone of the glycine-rich loop on the N-lobe and the C-terminal end of the activation loop on the C-lobe. In the inactive crystal structure this distance is only about 3 Å shorter than in the active-state crystal structure, but in the inactive simulations the lobes close together by another 4 Å (Fig 7). In the active simulations they remain open to facilitate substrate binding. Fig 7 shows that the groove between the N- and C-lobes closes more in the K377-ubiquitinated simulations than in the control simulations of the active state, with a Wilcoxon rank sum test p-value of 7.1x10^-3^. Starting from the inactive structure, ubiquitination at K476 or K377 leads to a substrate-binding groove that remains more open than the control (K476 p-value = 1.1x10^-5^, K377 p-value = 6.3x10^-11^ both using the Wilcoxon rank sum test and comparing to the inactive control). While the K476-ubiquitinated structure sampled a C-helix conformation closer to the active structure as well as sampling a more open binding groove, the open binding groove was the only large conformational difference for K377-ubiquitinated ZAP-70 starting from the inactive state. Overall, our simulations suggest that an addition of ubiquitin to ZAP-70 at K476 has the opposite effect of ubiquitination at K377, stabilizing the kinase active conformation rather than disrupting it.

It is possible that on a timescale longer than that of our MD simulations, the conformational change induced by ubiquitination of the active structure at K377 would become more pronounced and that the K377-ubiquitinated ZAP-70 would adopt the same conformational ensemble as the inactive kinase. This would probably require that the K369-E386 salt bridge be broken so that the C-helix could move farther away from the rest of the N-lobe. Alternatively, ubiquitination at K377 may not fully disrupt the ZAP-70 active state structure, but merely destabilize it. Either way, our simulations clearly lead to the hypothesis that ubiquitination of ZAP-70 could negatively affect kinase activity through a conformational change, involving both the C-helix and the orientations of the N- and C-terminal kinase lobes. We also observe that K476-ubiquitination stabilizes the ZAP-70 active structure, potentially improving kinase activity.

#### The conformational changes are particular to ubiquitination

**Fig 8:**
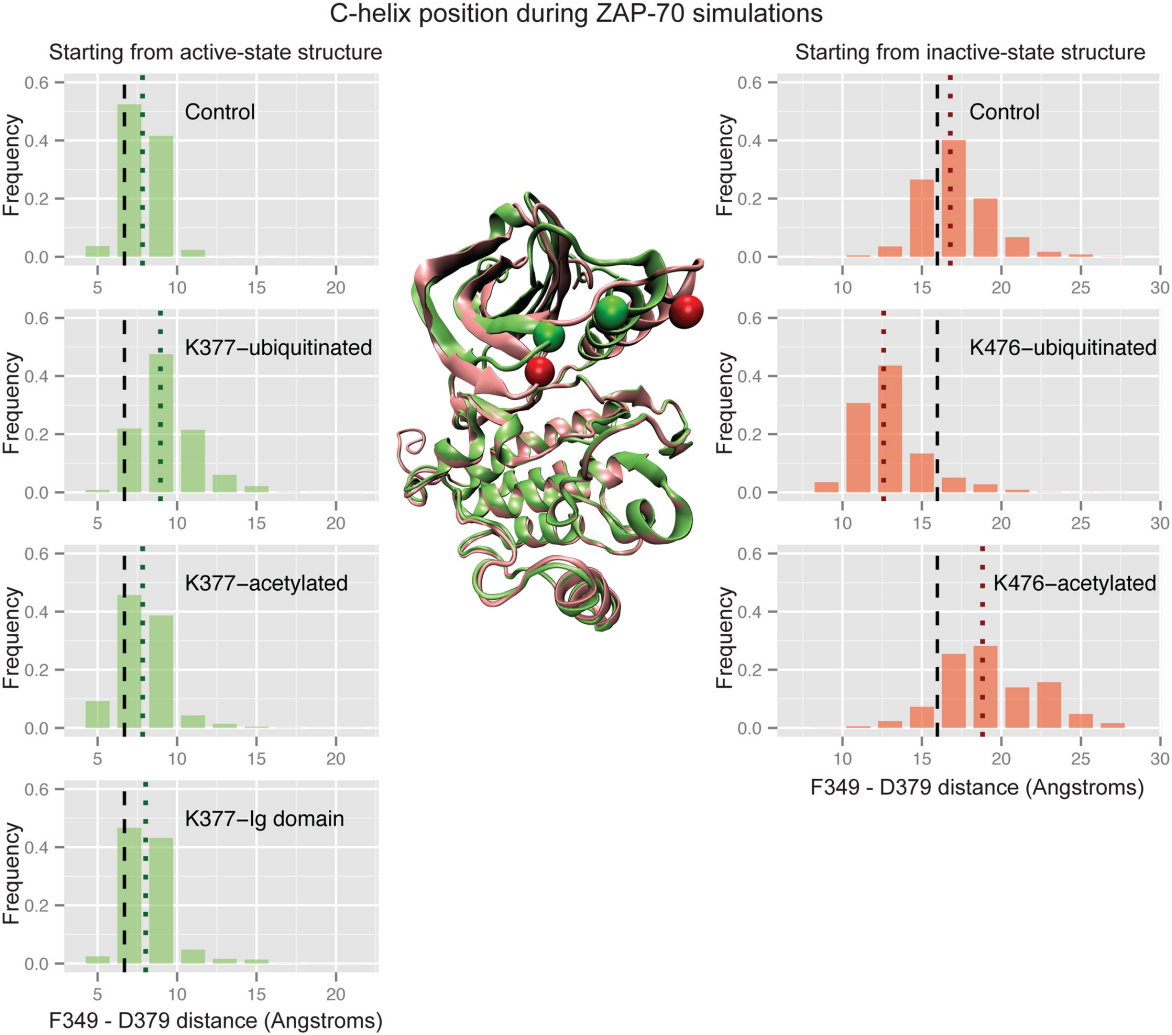
Acetylation and Ig domain attachment do not affect ZAP-70 C-helix position. Histograms of the distance between the F349 Cα atom and the D379 Cβ atom during ZAP-70 control simulations, K377-monoubiquitinated simulations, K377-acetylated simulations, and simulations with an Ig domain attached at K377, all started from the active state structure (left), as well as control, K476-monoubiquitinated, and K476-acetylated simulations started from the inactive, autoinhibited structure (right). The vertical dashed black lines indicate the distance observed in the starting crystal structure, while the dotted colored lines indicate the median value for each histogram. The histogram is created from combining all structures sampled in each of the 32 independent simulations with snapshots every picosecond. In the center, residues F349 and D379 are shown as spheres on the overlaid ZAP-70 active (green) and inactive (red) crystal structures.

**Fig 9:**
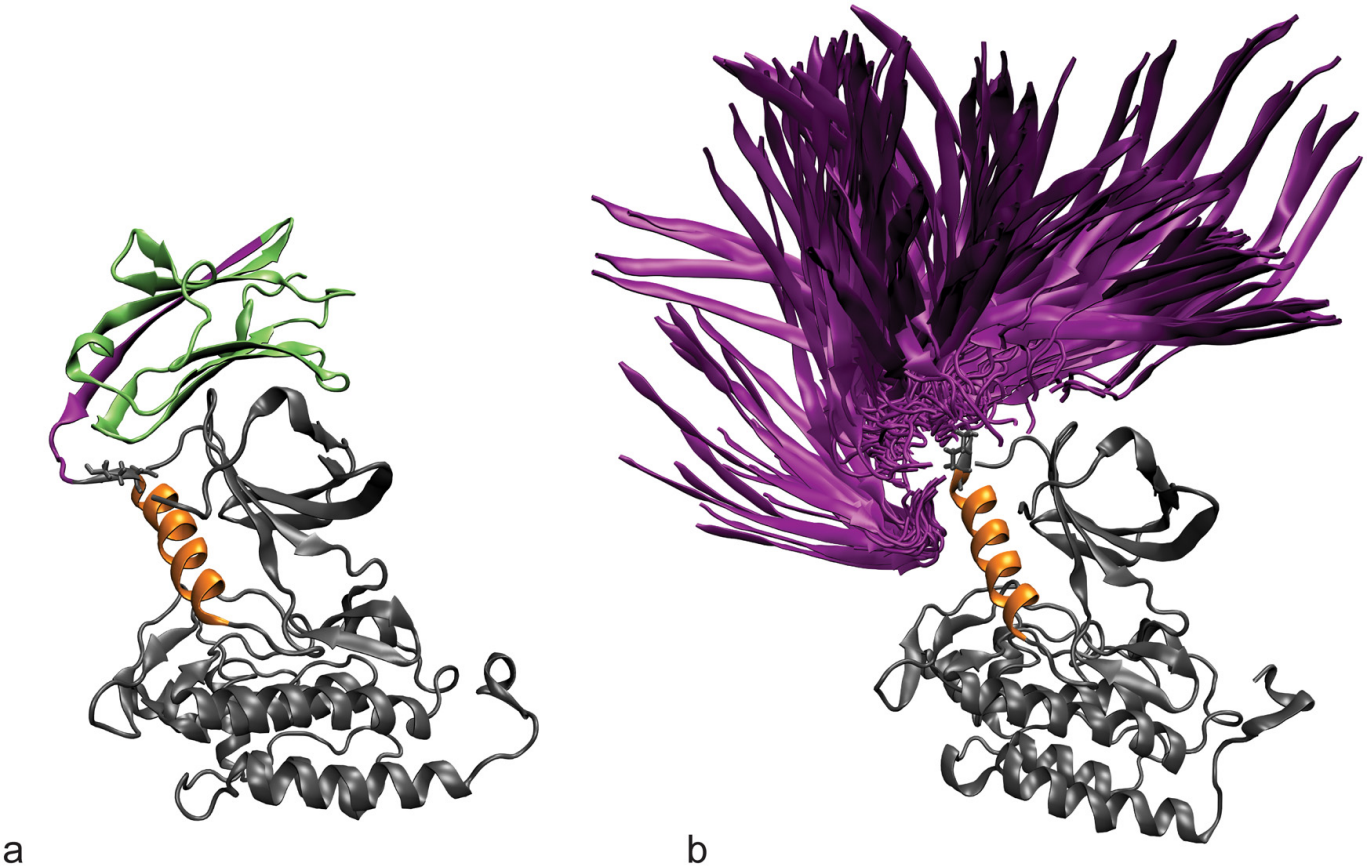
Ig domain sampling. **(a)** The starting structure for simulations of the active ZAP-70 kinase with an Ig domain attached at K377. The C-helix is shown in orange and the Ig domain molecule is shown in green. The C-terminal β-strand of the Ig domain is highlighted in purple. (b) The diversity of orientations that the Ig domain samples, starting from the K377 active structure with Ig domain attached, is summarized by showing only the orientation of the C-terminal β-strand (purple) relative to the kinase, for snapshots separated by 1 ns.

To test whether other types of modifications to K377 would result in the same effect, we ran simulations with acetylation instead of ubiquitination. Ubiquitination neutralizes the lysine residue where it is attached, and if the affect on conformation were purely due to electrostatic interactions, we might see a similar outcome with an acetylated lysine. As shown in Fig 8, acetylation has no effect on C-helix position in the case of K377-acetylation starting from the active structure. For K476-acetylation starting from the inactive structure, the C-helix moves slightly farther away from the rest of the kinase domain, which is opposite to the effect of K476-ubiquitination. We also ran simulations starting from the active structure with an Immunoglobulin (Ig) domain attached at K377 instead of ubiquitin (Fig 9). This domain is similar to ubiquitin in terms of size and β-sheet content, and has a similar number of positively and negatively charged residues. We anticipated that the conformational effect of ubiquitination at K377 would not be sequence specific and that the Ig domain would also disrupt the active conformation of the ZAP-70 C-helix. In fact, the Ig domain does not have the same effect as ubiquitin, as shown in Fig 8. The Ig domain attached at K377, in the same way as ubiquitin, does not show any differences from the control simulations, therefore the disruptive effect of ZAP-70 K377-ubiquitination appears to be specific to the ubiquitin sequence or fold. Effects of acetylation and Ig domain attachment on other metrics are given in the supporting information (S3 and S4 Figs).

## Discussion

We find that many of the ubiquitination sites on human kinases are proteasome insensitive, and therefore likely not associated with protein degradation. We hypothesize that some of these proteasome insensitive ubiquitin modifications influence the conformation of the modified protein. A simple way for ubiquitination to affect the structure of a folded protein is to shift the equilibrium populations of two alternate states. For protein kinases, the active conformation is often in equilibrium with an inactive state. There are several important structural elements that must be in place for kinase activity, including the N-lobe catalytic lysine, the C-helix, and the substrate binding groove [48,111]. Many different inactive conformations are possible, since any disruption of the active structure will decrease function. Often, as is true for ZAP-70, the C-helix is a major site of kinase regulation [48,74]. In the crystal structure of the autoinhibited form of ZAP-70, the C-helix is swung out from the active conformation, but our simulations provide a more detailed picture, revealing that the C-helix is also less structured and more flexible in the inactive state. Partial unfolding of a kinase C-helix has also been observed in simulations of the epidermal growth factor receptor in the inactive state [112]. Ubiquitination could regulate kinase activity by affecting the conformation of this key helix.

### Ubiquitin is a site-specific kinase regulator

On the kinase domain, we observe that the glycine-rich loop area and the region N-terminal to the activation loop are preferentially ubiquitinated, as did Swaney *et al*. [61]. Phosphorylation hot spots also cluster to the activation loop and the glycine-rich loop, which are key regions for kinase regulation [60]. Ubiquitination at regions of the kinase structure associated with conformational regulation indicates that ubiquitin attachment could directly modulate kinase activity (whether or not this is the primary role of the ubiquitination). We expect only proteasome insensitive ubiquitination to be responsible for conformational regulation, however both proteasome sensitive and insensitive ubiquitination are enriched in these regions, as well as at conserved lysine residues and near phosphorylation hot spots, as reported previously for other proteins [58,61]. Although proteasome sensitive ubiquitination leads to degradation, these ubiquitin modifications may still be site-specific because proteases degrade substrates by unfolding them starting at the site of ubiquitination [113]. Adding a layer of complexity, the same site can be associated with proteasomal degradation on one kinase, and not on a different kinase or under different cellular conditions.

There are also specific sites that are particularly associated with proteasome-insensitive ubiquitination. The conservation of these ubiquitination sites, like ZAP-70 K476, across kinases indicates that ubiquitination plays an important functional role at these sites, which is likely not related to proteasomal degradation. We also found that most ubiquitinated kinases had multiple sites of ubiquitination, as was previously observed in global ubiquitination studies [8]. For some of these kinases, such as ZAP-70, several of these sites are not associated with proteasomal degradation. In fact, these different ubiquitination sites may differentially affect conformation of the same protein. Our MD studies of two different ubiquitinated constructs of the ZAP-70 kinase domain indicate that attachment of a single ubiquitin molecule to a surface lysine modifies the kinase structure and dynamics in a site specific manner. Ubiquitination at K377, near the C-helix, causes ZAP-70 to adopt conformations closer to the inactive state, while ubiquitination at K476, near the hinge region, results in a conformational ensemble more similar to the active state. On the basis of these simulations, we hypothesize that ubiquitination at different sites may differentially modulate kinase activity and/or interactions. Since K476 is a conserved ubiquitination site on kinases, it is also possible that other kinases will undergo similar conformational changes to ZAP-70 when ubiquitinated at this site.

Importantly, we recognize that incomplete sampling is a limitation of our MD results. Our simulations starting from the active and inactive ZAP-70 crystal structures are fundamentally the same since the autoinhibitory domains from the inactive crystal structure are removed in our simulations. The differences in ZAP-70 conformation that we observe are a result of different starting structures. The K369-E386 salt bridge that holds the C-helix in the active state is not broken in our active structure simulations and not formed in our inactive structure simulations. If the sampling were increased, we would observe these rare events and the ensembles sampled starting from the active and inactive states would converge. Because we have run many independent short simulations, we also do not sample motions that occur on long timescales. We ran a single long simulation for each of four ZAP-70 constructs (active control, active K377-ubiquitinated, inactive control, and inactive K476-ubiquitinated) to compare to our data combined from many short simulations (S5 and S6 Figs). In these long simulations we also do not observe breaking or forming of the K369-E386 salt bridge, but we do observe more opening of the substrate binding groove in the K476-ubiquitinated simulations starting from the inactive structure, indicating that this may be a motion that occurs on a longer timescale (S5 Fig). Overall we see that the data from a single long simulation has a narrower distribution than the data from many short simulations (S5 and S6 Figs), since the different time points from a single simulation are correlated. We therefore prefer many short simulations to a single long simulation for assessing the effects of ubiquitination on ZAP-70. Given total simulation times on the order of 100 ns, these short simulations provide better sampling of the true conformational distribution for ubiquitinated ZAP-70.

Having simulated only the kinase domain, we also cannot directly infer what the effects of ubiquitination are on the full-length ZAP-70. However, our simulations may be a reasonable approximation of the ZAP-70 kinase domain following TCR complex binding and the release of the SH2 domains from the kinase domain. After this release, the SH2 domains only interact with the kinase domain through a flexible linker, and the kinase domain is catalytically active when the SH2 domains are cleaved [81]. Another limitation of this study is the presence of only one ubiquitination molecule in the ZAP-70 simulations. *In vivo*, a polyubiquitin chain may be attached; however, we cannot distinguish the number of ubiquitin molecules or the linkage residue for polyubiquitination. Given this ambiguity, we chose to simulate monoubiquitinated ZAP-70, which is the simplest possible construct.

### Ubiquitin is a unique post-translational modification

Our simulations indicate that ubiquitination has the potential to regulate kinase structure and dynamics, leading us to compare its effects to other PTMs like phosphorylation. We found that kinase ubiquitination occurs more frequently on a folded domain than on unstructured linkers or tails, unlike phosphorylation, which occurs more in unstructured regions. Similar trends were reported by Wagner *et al*. and Hagai *et al*. in studies of ubiquitination across all protein families [62,63], and Duttler *et al*. found that ubiquitination of nascent protein chains also is not enriched in regions of intrinsic disorder [64]. Several studies have noted that within these structured domains, ubiquitination tends to occur on loops or flexible regions rather than helices, or, in particular, β-sheets [114–116]. Beltrao *et al*. also found that ~75% of phosphorylation sites, 40% of acetylation sites, and 45% of ubiquitination sites occur outside PFAM globular domains, after looking at all proteins in the PTMfunc database [60]. Focusing only on kinases, we found that ubiquitination occurs outside folded domains even more rarely, only ~30%. While phosphorylation on linkers and tails allows these regions of kinases to become ordered or disordered, affecting kinase conformation, ubiquitination likely regulates kinases by a different mechanism. Acetylation, like ubiquitination, occurs in structured regions, and in fact one of its roles may be to protect against ubiquitination. Wagner *et al*. found that 30% of acetylated lysines are also ubiquitinated, and these residues show a lower dependence on proteasome inhibitor [62]. However, while acetylated lysines show an enrichment in basic residues in the flanking sequence, flanking regions of ubiquitination sites are enriched in acidic aspartate residues [115,116]. Both acetylation and ubiquitination sites tend to be near hydrophobic residues, which may indicate that they are more buried and modifications at these sites could result in large conformational changes [116]. Based on the structural preferences of ubiquitination for folded domains of kinases, we expect it to play a different role in kinase regulation than phosphorylation.

Both phosphorylation and acetylation primarily affect protein conformation by perturbing the energy landscape electrostatically. For example, simulations of phosphorylated kinases reveal how the addition of a negatively charged phosphate group on the activation loop can stabilize a protein conformation that is aligned for kinase activity, or in some cases directly stabilize the correct orientation of the substrate side chain [65]. The few molecular dynamics studies of lysine acetylation suggest that, analogous to phosphorylation, its primary effect may be driven by electrostatics, i.e., neutralization of the positive charge on the Lys side chain [70,71]. Like acetylation, ubiquitination neutralizes the positive charge on the lysine residue. However, in our simulations acetylation does not have the same affect as ubiquitination on conformation at either ZAP-70 site. Unlike acetylation and phosphorylation, the effect of ubiquitination on the kinase energy landscape is not primarily electrostatic. Ubiquitin is much larger than the acetyl group and this bulky group may have significant entropic consequences including solvent effects.

### Allosteric effects of ubiquitination on kinase conformation

We observe that ubiquitination at residues that are not critical to kinase activity may allosterically regulate the kinase by changing its overall conformation. In the case of ZAP-70 ubiquitination, this appears to be mediated by the position and structure of the C-helix and possibly the peptide-binding groove. Previous work by McClendon *et al*. used MD simulations of PKA and the mutual information between residues to reveal a network of correlated motions that could mediate long-distance allosteric coupling in kinases [117]. There are several ways that ubiquitin may be perturbing these correlated motions to alter kinase conformation. Our simulations of ubiquitinated ZAP-70 show that the ubiquitin moiety is attached flexibly to the kinase and samples many orientations (Fig 4). This supports earlier observations of multiple ubiquitin orientations for the ubiquitinated Ras protein from NMR and computational models [24], and indicates that the effects of ubiquitin may be entropic.

Molecular dynamics simulations of ubiquitinated proteins by Hagai and Levy have characterized the effect of ubiquitination on thermodynamic stability of a protein [118]. They observe a decrease in protein stability upon ubiquitination that they attribute to entropic stabilization of the unfolded state of the protein. They conclude that destabilization, along with local unfolding at the ubiquitination site, may facilitate protein unfolding prior to degradation. In our K377-ubiquitinated ZAP-70 simulations, we observe partial unfolding of the C-helix, which also moves away from the rest of the N-lobe (Figs 5 & 6). Entropic stabilization of the locally unfolded state may explain this partial C-helix unfolding.

**Fig 10:**
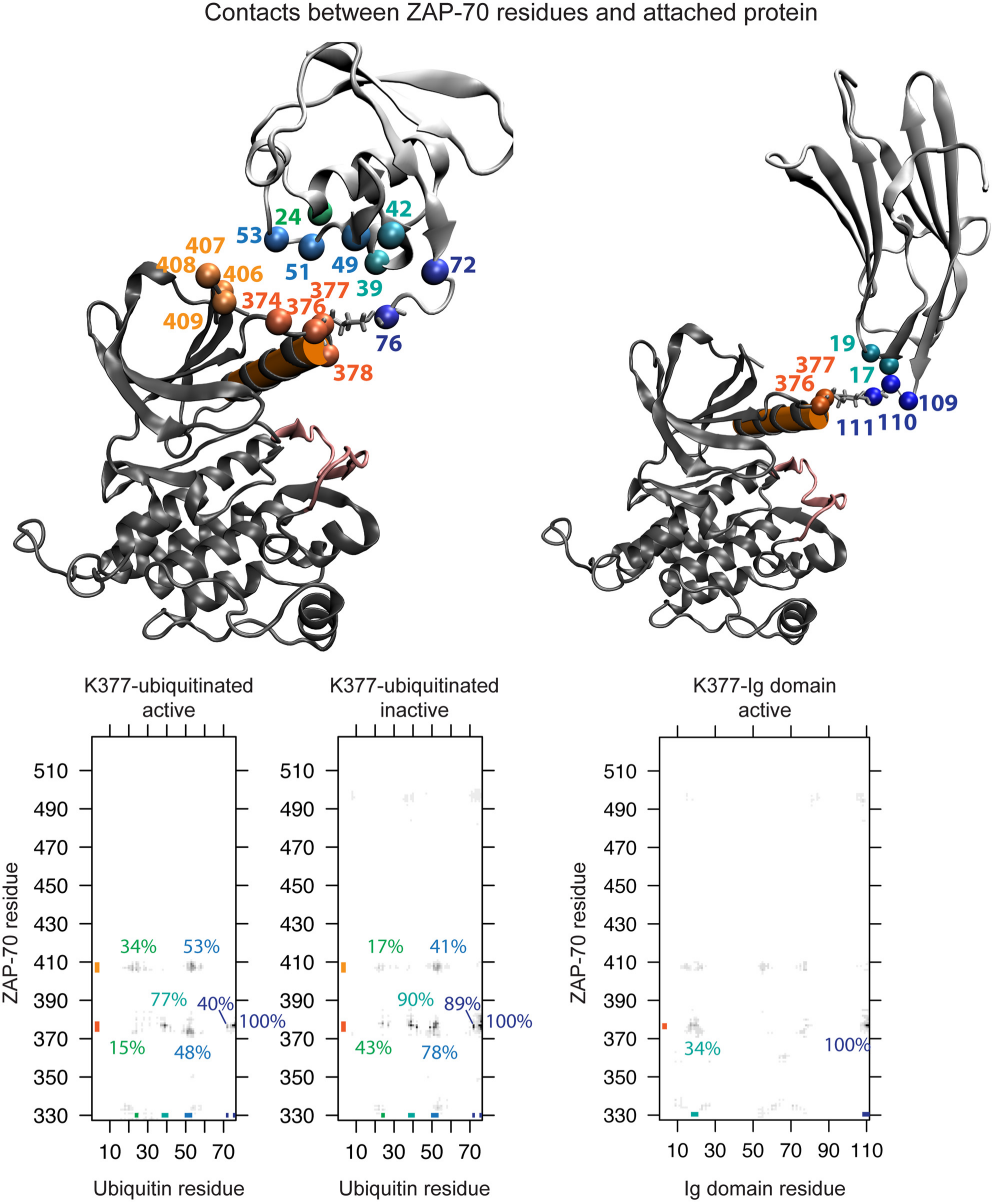
ZAP-70 contacts with ubiquitin or the Ig domain. Contact maps showing the ZAP-70 sequence vs. the ubiquitin sequence for K377 ubiquitination starting from the active-state structure and inactive-state structure, and a contact map showing the ZAP-70 sequence vs. the Ig domain sequence for K377 Ig domain attachment starting from the active-state structure. The darkness of the pixel indicates the percent of snapshots from the simulation where these two residues are in contact. A contact is defined as a distance of less than 10 Å between residue centers of mass. In the structures shown above, the residues that are frequently involved in contacts are colored and labeled, both on ZAP-70 and the ubiquitin and Ig domain moieties. The attached protein is shown in white, while the ZAP-70 structure is shown in dark gray. The corresponding colors are shown next to the axes on the contact maps. The contact regions with the highest frequency are also labeled with their frequencies on the contact maps. The color of these labels corresponds to the color of the ubiquitin or Ig domain residues involved.

We were surprised to observe that ubiquitin attachment at K377 leads to C-helix disruption, while the attachment of the similarly sized Ig domain at K377 (Fig 9) does not significantly disrupt the C-helix (Fig 8). This indicates that the specific sequence or shape of the ubiquitin molecule plays a role in disrupting the ZAP-70 C-helix structure. Fig 10 shows ZAP-70 contacts with both the ubiquitin and Ig-domain molecules when attached at K377. The contacts with ubiquitin are present in much more of the simulated ensemble than the contacts with the Ig domain. Some of the ubiquitin contacts likely result from forming hydrogen bonds, but ion pairs also transiently form between ubiquitin and the kinase. For K377-ubiquitinated ZAP-70, there are contacts between E376 and both R72 and R42 on ubiquitin, especially in the inactive state simulations. Interestingly, R72 and R42 are near the ubiquitin “hydrophobic patch” that is the site of recognition by ubiquitin binding domains [119], although the key hydrophobic patch residue, I44, does not contact the kinase. In the simulations with the Ig domain attached at residue 377, we do not observe any ion pair interactions between the two proteins, nor are there significant hydrophobic contacts formed. This may explain why no conformational change occurs in simulations with the Ig domain attached to ZAP-70.

Electrostatic interactions, like what we observe between ZAP-70 and ubiquitin, are also present in the ubiquitinated structure of PCNA. K164-ubiquitinated PCNA adopts a different conformation from SUMOylated PCNA due to specific electrostatic interactions with ubiquitin R42, K48, E51, D58, and D39 [31]. In the SUMOylated structure, however there is electrostatic repulsion between two negatively charged surfaces, resulting in an extended conformation with few contacts [31], as we observe with Ig domain. Like our simulations, the studies of PCNA ubiquitination and SUMOylation only included one ubiquitin or SUMO molecule. Since ubiquitin moieties that are part of a polyubiquitin chain are known to interact with each other, there could be competition for the interactions with ZAP-70 if it were polyubiquitinated. However, the conformational effects might also be exaggerated with the presence of multiple ubiquitin moieties.

**Fig 11:**
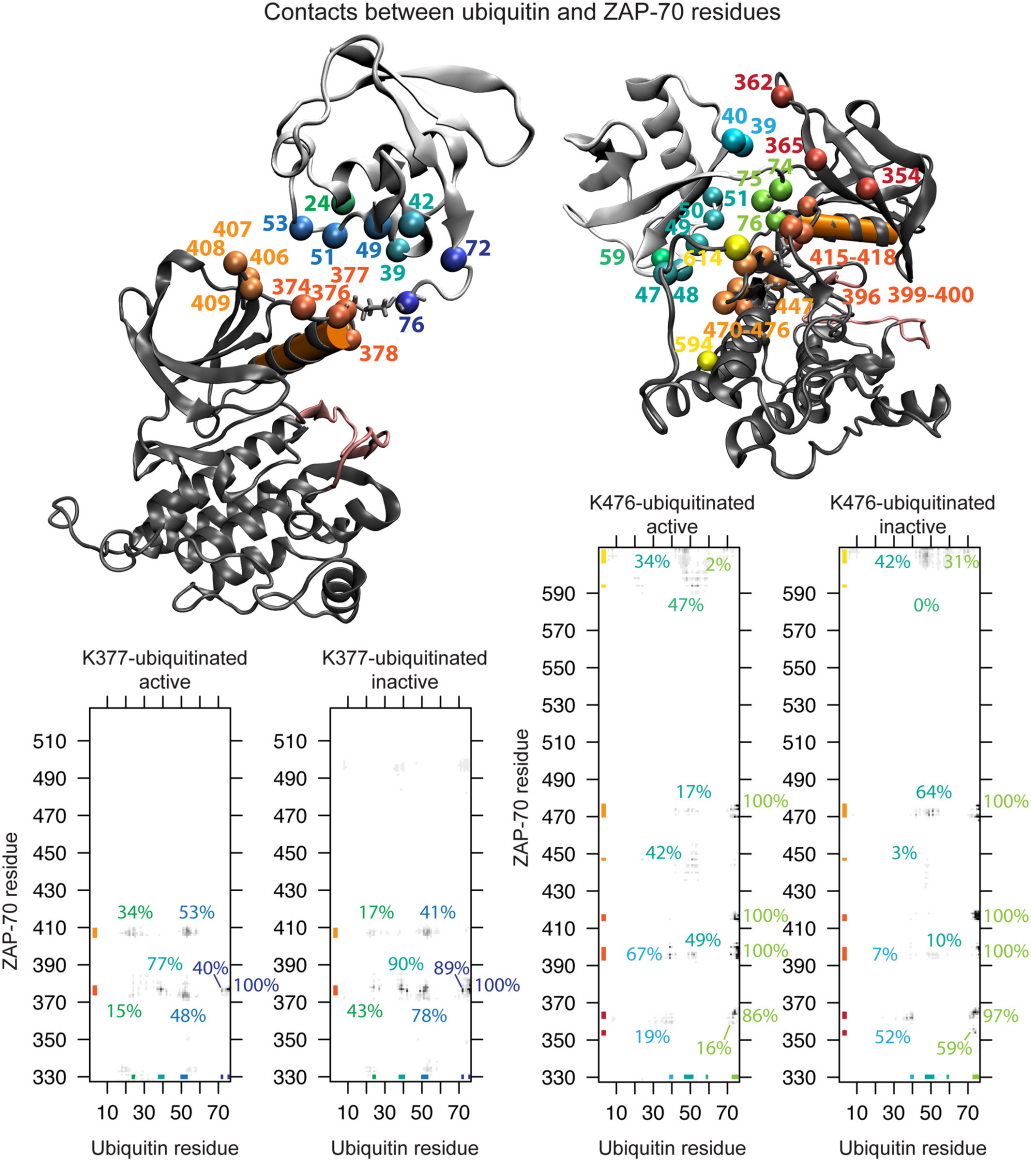
Ubiquitin-ZAP-70 contacts. Contact maps showing the ZAP-70 sequence vs. the ubiquitin sequence for K377 ubiquitination starting from the active-state structure and inactive-state structure, and K476 ubiquitination starting from the active-state structure and inactive-state structure. The darkness of the pixel indicates the percent of snapshots from the simulation where these two residues are in contact. A contact is defined as a distance of less than 10 Å between residue centers of mass. In the ubiquitinated structures shown above, the residues that are frequently involved in contacts are colored and labeled, both on ubiquitin and ZAP-70. The ubiquitin moiety is shown in white, while the ZAP-70 structure is shown in dark gray. The corresponding colors are shown next to the axes on the contact maps. The contact regions with the highest frequency are also labeled with their frequencies on the contact maps. The color of these labels corresponds to the color of the ubiquitin residues involved.

Specific interactions between ubiquitin and ZAP-70 in the K476-ubiquitinated simulations are shown in Fig 11. In the K476-ubiquitinated simulations, both E415 and D365 on ZAP-70 interact with R74 on ubiquitin, and ZAP-70 R594 contacts ubiquitin E51. The ZAP-70 C-terminal F614 also interacts with hydrophobic ubiquitin residues. However, while K377-ubiquitinated ZAP-70 ionic interactions could directly perturb the C-helix linker, a more complex mechanism is likely at play for K476 ubiquitination, since ubiquitin does not directly contact the C-helix in this construct. Hagai and Levy note that the thermodynamic effects of ubiquitination vary depending on the attachment site [118]. Atomistic MD simulations also show that ubiquitination of Ubc7, an E2 enzyme, decreases the flexibility of certain regions of Ubc7 although there were no specific direct interactions between ubiquitin and the E2 [120]. We observe similar stabilization of the C-helix with ubiquitination at K476, also in the absence of any stable interactions between ubiquitin and ZAP-70. The Ubc7 simulations also show that the strongest effect occurred with K48-linked tetraubiquitination at known sites of degradative ubiquitination [120], indicating that polyubiquitination could have an even larger effect on ZAP-70 conformation than monoubiquitination.

**Fig 12:**
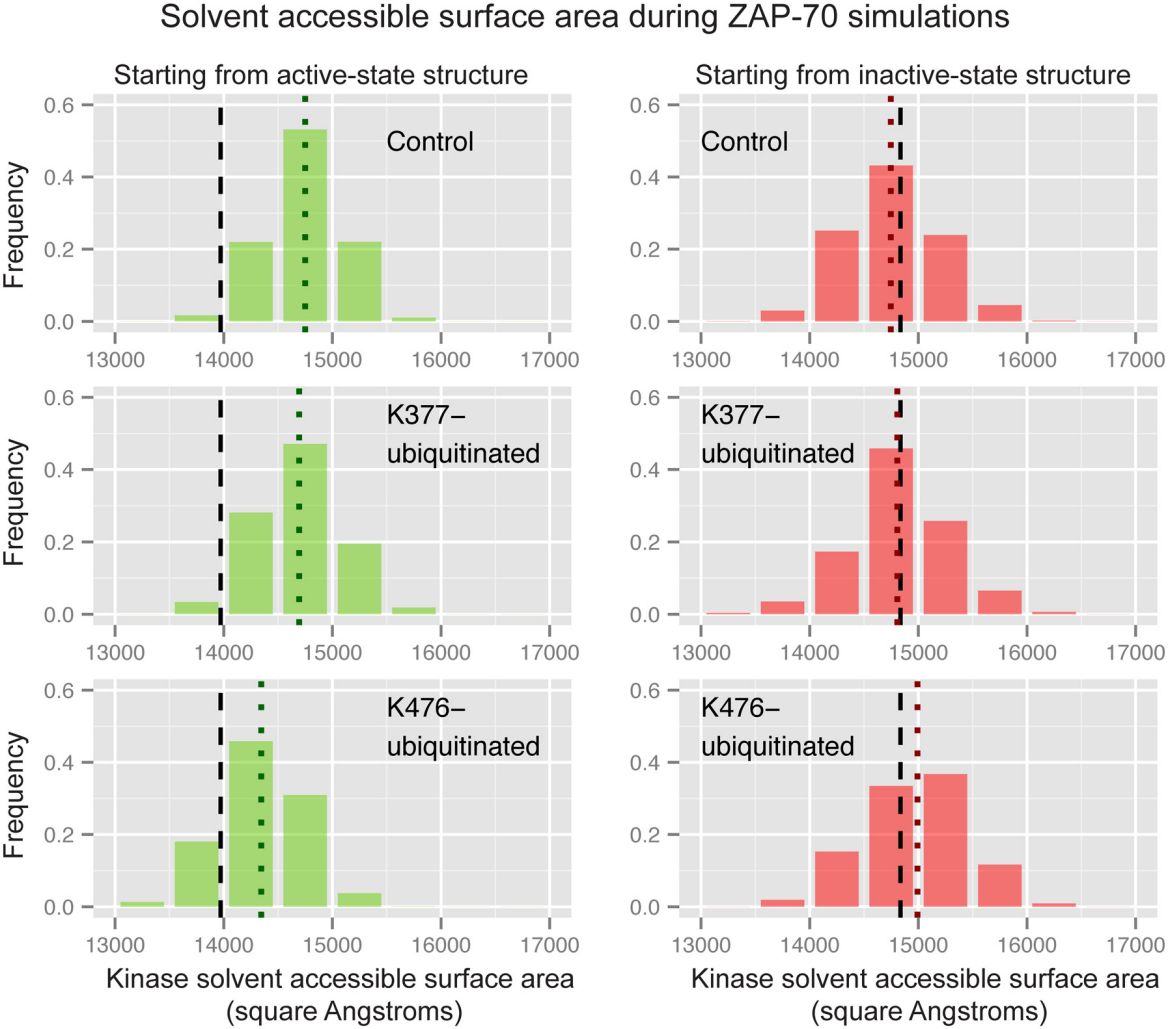
ZAP-70 kinase domain solvent accessible surface area. Histogram of the solvent accessible surface area during the control, K377-ubiquitinaed, and K476-ubiquitinated simulations starting from the active state structure (left) and inactive structure (right). The vertical dashed black lines indicate the surface area for the starting crystal structure, while the dotted colored lines indicate the median value for each histogram.

The K476 ubiquitination site is farther from the C-helix than K377, yet somehow the presence of ubiquitin shifts the conformational equilibrium toward an active kinase structure with a stable folded C-helix that remains close to the rest of the N-lobe. Coupling between the activation loop and the C-helix can allow communication between the active site and the back of the kinase, where K476 is located [48]; however, we could not find any specific residues between K476 and the active site that were perturbed by the presence of ubiquitin. Another possible mechanism involves the bulky ubiquitin modifying the interaction between the protein and solvent. In the simulations of unmodified ZAP-70 as well as all K377-ubiquitinated simulations, the solvent accessible surface area of the kinase domain remains similar (Fig 12). In contrast, K476-ubiquitination modifies the exposed surface area, decreasing it slightly when starting from the active state, and increasing it significantly when starting from the inactive state (Fig 12). These results imply a net decrease in solvent accessible surface area from the inactive to active state when K476 is ubiquitinated. Thus, ubiquitin attachment may indirectly impact protein structure and dynamics by modulating solvation, which could involve both enthalpic interactions between water and the protein, and increases in water entropy accompanying decreased solvent exposure. In the case of polyubiquitination, the attached moiety would be even larger, possibly increasing or modifying the solvent-mediated effects of ubiquitination on conformation.

Our study suggests that ubiquitination may regulate kinases in a manner unrelated to degradation, and that this regulation could result from site-specific changes in conformational dynamics. Simulations of ZAP-70 predict that ubiquitination at K377 or K476 could respectively reduce or increase kinase activity. Ubiquitination may also affect conformation in other protein families, and future work should explore the role of ubiquitin in non-degradative signaling, including further investigation of the biophysical effects of ubiquitination.

## Supporting Information

S1 Table Details of simulation solvent box for ZAP-70 constructs. (PDF)

S2 Table HIV dependence of ubiquitination sites identified in Jurkat and HEK293 cells. (PDF)

S3 Table P-value statistics testing the hypothesis that simulation metrics have the same distribution as the unmodified control simulations started from the same ZAP-70 crystal structure. (PDF)

S4 Table P-value statistics testing the hypothesis that simulation metrics have the same distribution as the unmodified control simulations started from the same ZAP-70 crystal structure. Using the medians of each simulation as the data.
(PDF)

S1 Fig Ubiquitinated ending structures. Example ending conformation from active K377-ubiquitinated simulations superimposed on the ZAP-70 active crystal structure (a) and from inactive K476-ubiquitinated simulations superimposed on the ZAP-70 inactive crystal structure (b). The crystal structure is shown in grey, C-helix residues are highlighted in green (a) or red (b), and the ubiquitin molecule is shown in cyan. (a) The F349Cα-D379Cβ distance = 14.9 Å. (b) The F349Cα-D379Cβ distance = 7.6 Å.
(TIF)

S2 Fig Known substrates of HIV ubiquitination are detected in HEK293 cells expressing HIV. The sum of MS1 intensities of all ubiquitinated peptides are plotted for known substrates of HIV-mediated ubiquitination CD4 (a) and APOBEC3C (b) in response to doxycycline-induced expression of HIV.
(TIFF)

S3 Fig Acetylation and Ig domain attachment do not affect ZAP-70 C-helix order. Histograms of the number of residues in the C-helix (380–393) that retain the helical conformation, using the DSSP definition, during ZAP-70 control simulations, K377-monoubiquitinated simulations, K377-acetylated simulations, and simulations with the Ig domain attached at K377, all started from the active state structure (left), as well as control, K476-monoubiquitinated, and K476-acetylated simulations started from the inactive, autoinhibited structure (right). The vertical dashed black lines indicate the distance observed in the starting crystal structure, while the dotted colored lines indicate the median value for each histogram. The histogram is created from combining all structures sampled in each of the 32 independent simulations with snapshots every picosecond. In the center, the C-helix is highlighted on the ZAP-70 active crystal structure (green) and an overlaid structure from the inactive conformation simulations (red) where the C-helix is mostly unraveled (only 6 residues have helical secondary structure).
(TIF)

S4 Fig Acetylation and Ig domain attachment do not affect ZAP-70 groove closure. Histograms of the distance between the N348 Cα atom and the W501 Cα atom during ZAP-70 control simulations, K377-monoubiquitinated simulations, K377-acetylated simulations, and simulations with the Ig domain attached at K377, all started from the active state structure (left), as well as control, K476-monoubiquitinated, and K476-acetylated simulations started from the inactive, autoinhibited structure (right). The vertical dashed black lines indicate the distance observed in the starting crystal structure, while the dotted colored lines indicate the median value for each histogram. The histogram is created from combining all structures sampled in each of the 32 independent simulations with snapshots every picosecond. In the center, residues N348 and W501 are highlighted on the overlaid ZAP-70 active (green) and inactive (red) crystal structures.
(TIF)

S5 Fig Results of single long simulation started from the active state crystal structure, with and without ubiquitination at K377. Histograms of the distance between the F349 Cα atom and the D379 Cβ atom (a), the number of residues in the C-helix (380–393) that retain the helical conformation, using the DSSP definition (b), and the distance between the N348 Cα atom and the W501 Cα atom (c). Each of these is plotted for the set of 32 short control simulations, the single 100-ns control simulation, the set of 32 short K377-ubiquitinated simulations, and the single 560-ns K377-ubiquitinated simulation. All of these simulations started from the ZAP-70 active state crystal structure. The vertical dashed black lines indicate the distance observed in the starting crystal structure, while the dotted colored lines indicate the median value for each histogram. The histogram is created by combining all structures sampled in each of the 32 independent simulations (or single long simulation) with snapshots every picosecond.
(TIF)

S6 Fig Results of single long simulation started from the inactive state crystal structure, with and without ubiquitination at K476. Histograms of the distance between the F349 Cα atom and the D379 Cβ atom (a), the number of residues in the C-helix (380–393) that retain the helical conformation, using the DSSP definition (b), and the distance between the N348 Cα atom and the W501 Cα atom (c). Each of these is plotted for the set of 32 short control simulations, the single 100-ns control simulation, the set of 32 short K476-ubiquitinated simulations, and the single 560-ns K476-ubiquitinated simulation. All of these simulations started from the ZAP-70 inactive state crystal structure. The vertical dashed black lines indicate the distance observed in the starting crystal structure, while the dotted colored lines indicate the median value for each histogram. The histogram is created by combining all structures sampled in each of the 32 independent simulations (or single long simulation) with snapshots every picosecond.
(TIF)

S1 Dataset Protein abundance data from Jurkat cells for ubiquitin, ISG15, NEDD8, several interferon induced proteins, and BST2, which are known to be degraded upon HIV infection. Table of protein abundance fold-changes and adjusted p-values as calculated by MSstats (see Methods) for ubiquitin, ISG15, NEDD8, and select interferon-induced proteins.
(XLSX)

S2 Dataset All ubiquitination sites from combined Jurkat cell dataset and PTMfunc database. There is a row for each ubiquitination site and the columns tell the name of the protein ubiquitinated, the residue number of the modification, the corresponding aligned residue on PKA (if there is one), the corresponding aligned residue on ZAP-70 (if there is one), whether the site is proteasome dependent or independent (if known), whether the site is on the kinase domain, whether the site is on any folded protein domain, whether the modification shows any dependence on HIV infection (if known), whether the abundance of the corresponding peptide shows any dependence on HIV infection (if known), whether the ubiquitination site is near a phosphorylation site, whether the ubiquitination site is near a phosphorylation hot spot as designated by the PTMfunc database, and the source of the modification (Jurkat cell experiment, PTMfunc database, or both).
(XLSX)

S3 Dataset Ubiquitination sites from HEK293 cell dataset. There is a row for each ubiquitination site and the columns tell the name of the protein ubiquitinated, the residue number of the modification, the corresponding aligned residue on PKA (if there is one), the corresponding aligned residue on ZAP-70 (if there is one), whether the site is proteasome dependent or independent (if known), whether the site is on the kinase domain, whether the site is on any folded protein domain, whether the modification shows any dependence on HIV infection (if known), whether the ubiquitination site is near a phosphorylation site, whether the ubiquitination site is near a phosphorylation hot spot as designated by the PTMfunc database.
(XLSX)

S4 Dataset Jurkat cell kinase ubiquitination data. Log2-fold-changes (L2FC) and adjusted p-values as calculated by MSstats (see Materials and Methods) for ubiquitination sites in Jurkat cells infected with HIV. UniProt accessions, gene names, descriptions, and modified site positions are indicated; multiple accessions separated by semi-colons indicate sites for which peptide sequences were identical between multiple UnirProt entries. L2FC and adjusted p-values are indicated for the following comparisons: HIV-infected vs. uninfected cells, HIV-infected and MG-132-treated vs uninfected and MG-132-treated cells, and uninfected MG-132-treated and uninfected untreated cells.
(XLS)

S5 Dataset HEK293 cell kinase ubiquitination data. Log2-fold-changes (L2FC) and adjusted p-values as calculated by MSstats (see Materials and Methods) for ubiquitination sites in HEK293 cells engineered to express HIV by a tetracycline-responsive promoter. UniProt accessions, gene names, descriptions, and modified site positions are indicated; multiple accessions separated by semi-colons indicate sites for which peptide sequences were identical between multiple UnirProt entries. L2FC and adjusted p-values are indicated for the following comparisons: all doxycycline-treated vs. all untreated cells (Dox all v Mock all), doxycycline-treated MG-132-treated vs. doxycycline-treated cells (Dox MG v Dox), doxycycline-treated MG-132-treated vs. MG-132-treated cells (Dox MG v Mock MG), and doxycycline-treated vs. untreated cells (Dox v Mock).
(XLS)

S6 Dataset ZAP-70 active simulation F349 Cα—D379 Cβ distance file. The distance between the ZAP-70 F349 Cα atom and the D379 Cβ atom at each picosecond in the simulations is printed in Angstroms. Data from each of the 32 10-ns simulations is combined into one file.
(BZ2)

S7 Dataset ZAP-70 inactive simulation F349 Cα—D379 Cβ distance file. The distance between the ZAP-70 F349 Cα atom and the D379 Cβ atom at each picosecond in the simulations is printed in Angstroms. Data from each of the 32 10-ns simulations is combined into one file.
(BZ2)

S8 Dataset ZAP-70 K377-ubiquitinated active simulation F349 Cα—D379 Cβ distance file. The distance between the ZAP-70 F349 Cα atom and the D379 Cβ atom at each picosecond in the simulations is printed in Angstroms. Data from each of the 32 10-ns simulations is combined into one file.
(BZ2)

S9 Dataset ZAP-70 K377-ubiquitinated inactive simulation F349 Cα—D379 Cβ distance file. The distance between the ZAP-70 F349 Cα atom and the D379 Cβ atom at each picosecond in the simulations is printed in Angstroms. Data from each of the 32 10-ns simulations is combined into one file.
(BZ2)

S10 Dataset ZAP-70 K476-ubiquitinated active simulation F349 Cα—D379 Cβ distance file. The distance between the ZAP-70 F349 Cα atom and the D379 Cβ atom at each picosecond in the simulations is printed in Angstroms. Data from each of the 32 10-ns simulations is combined into one file.
(BZ2)

S11 Dataset ZAP-70 K476-ubiquitinated inactive simulation F349 Cα—D379 Cβ distance file. The distance between the ZAP-70 F349 Cα atom and the D379 Cβ atom at each picosecond in the simulations is printed in Angstroms. Data from each of the 32 10-ns simulations is combined into one file.
(BZ2)

S12 Dataset ZAP-70 K377-acetylated active simulation F349 Cα—D379 Cβ distance file. The distance between the ZAP-70 F349 Cα atom and the D379 Cβ atom at each picosecond in the simulations is printed in Angstroms. Data from each of the 32 10-ns simulations is combined into one file.
(BZ2)

S13 Dataset ZAP-70 K476-acetylated inactive simulation F349 Cα—D379 Cβ distance file. The distance between the ZAP-70 F349 Cα atom and the D379 Cβ atom at each picosecond in the simulations is printed in Angstroms. Data from each of the 32 10-ns simulations is combined into one file.
(BZ2)

S14 Dataset ZAP-70 K377-Ig domain active simulation F349 Cα—D379 Cβ distance file. The distance between the ZAP-70 F349 Cα atom and the D379 Cβ atom at each picosecond in the simulations is printed in Angstroms. Data from each of the 32 10-ns simulations is combined into one file.
(BZ2)

S15 Dataset ZAP-70 100-ns active simulation F349 Cα—D379 Cβ distance file. The distance between the ZAP-70 F349 Cα atom and the D379 Cβ atom at each picosecond in the simulations is printed in Angstroms.
(BZ2)

S16 Dataset ZAP-70 100-ns inactive simulation F349 Cα—D379 Cβ distance file. The distance between the ZAP-70 F349 Cα atom and the D379 Cβ atom at each picosecond in the simulations is printed in Angstroms.
(BZ2)

S17 Dataset ZAP-70 560-ns K377-ubiquitinated active simulation F349 Cα—D379 Cβ distance file. The distance between the ZAP-70 F349 Cα atom and the D379 Cβ atom at each picosecond in the simulations is printed in Angstroms.
(BZ2)

S18 Dataset ZAP-70 560-ns K476-ubiquitinated inactive simulation F349 Cα—D379 Cβ distance file. The distance between the ZAP-70 F349 Cα atom and the D379 Cβ atom at each picosecond in the simulations is printed in Angstroms.
(BZ2)

S19 Dataset ZAP-70 active simulation C-helix secondary structure file. The DSSP secondary structure of each ZAP-70 residue at each picosecond in the simulations is printed. Data from each of the 32 10-ns simulations is combined into one file.
(BZ2)

S20 Dataset ZAP-70 inactive simulation C-helix secondary structure file. The DSSP secondary structure of each ZAP-70 residue at each picosecond in the simulations is printed. Data from each of the 32 10-ns simulations is combined into one file.
(BZ2)

S21 Dataset ZAP-70 K377-ubiquitinated active simulation C-helix secondary structure file. The DSSP secondary structure of each ZAP-70 residue at each picosecond in the simulations is printed. Data from each of the 32 10-ns simulations is combined into one file.
(BZ2)

S22 Dataset ZAP-70 K377-ubiquitinated inactive simulation C-helix secondary structure file. The DSSP secondary structure of each ZAP-70 residue at each picosecond in the simulations is printed. Data from each of the 32 10-ns simulations is combined into one file.
(BZ2)

S23 Dataset ZAP-70 K476-ubiquitinated active simulation C-helix secondary structure file. The DSSP secondary structure of each ZAP-70 residue at each picosecond in the simulations is printed. Data from each of the 32 10-ns simulations is combined into one file.
(BZ2)

S24 Dataset ZAP-70 K476-ubiquitinated inactive simulation C-helix secondary structure file. The DSSP secondary structure of each ZAP-70 residue at each picosecond in the simulations is printed. Data from each of the 32 10-ns simulations is combined into one file.
(BZ2)

S25 Dataset ZAP-70 K377-acetylated active simulation C-helix secondary structure file. The DSSP secondary structure of each ZAP-70 residue at each picosecond in the simulations is printed. Data from each of the 32 10-ns simulations is combined into one file.
(BZ2)

S26 Dataset ZAP-70 K476-acetylated inactive simulation C-helix secondary structure file. The DSSP secondary structure of each ZAP-70 residue at each picosecond in the simulations is printed. Data from each of the 32 10-ns simulations is combined into one file.
(BZ2)

S27 Dataset ZAP-70 K377-Ig domain active simulation C-helix secondary structure file. The DSSP secondary structure of each ZAP-70 residue at each picosecond in the simulations is printed. Data from each of the 32 10-ns simulations is combined into one file.
(BZ2)

S28 Dataset ZAP-70 100-ns active simulation C-helix secondary structure file. The DSSP secondary structure of each ZAP-70 residue at each picosecond in the simulations is printed.
(BZ2)

S29 Dataset ZAP-70 100-ns inactive simulation C-helix secondary structure file. The DSSP secondary structure of each ZAP-70 residue at each picosecond in the simulations is printed.
(BZ2)

S30 Dataset ZAP-70 560-ns K377-ubiquitinated active simulation C-helix secondary structure file. The DSSP secondary structure of each ZAP-70 residue at each picosecond in the simulations is printed.
(BZ2)

S31 Dataset ZAP-70 560-ns K476-ubiquitinated inactive simulation C-helix secondary structure file. The DSSP secondary structure of each ZAP-70 residue at each picosecond in the simulations is printed.
(BZ2)

S32 Dataset ZAP-70 active simulation N348 Cα—W501 Cα distance file. The distance between the ZAP-70 N348 Cα atom and the W501 Cα atom at each picosecond in the simulations is printed in Angstroms. Data from each of the 32 10-ns simulations is combined into one file.
(BZ2)

S33 Dataset ZAP-70 inactive simulation N348 Cα—W501 Cα distance file. The distance between the ZAP-70 N348 Cα atom and the W501 Cα atom at each picosecond in the simulations is printed in Angstroms. Data from each of the 32 10-ns simulations is combined into one file.
(BZ2)

S34 Dataset ZAP-70 K377-ubiquitinated active simulation N348 Cα—W501 Cα distance file. The distance between the ZAP-70 N348 Cα atom and the W501 Cα atom at each picosecond in the simulations is printed in Angstroms. Data from each of the 32 10-ns simulations is combined into one file.
(BZ2)

S35 Dataset ZAP-70 K377-ubiquitinated inactive simulation N348 Cα—W501 Cα distance file. The distance between the ZAP-70 N348 Cα atom and the W501 Cα atom at each picosecond in the simulations is printed in Angstroms. Data from each of the 32 10-ns simulations is combined into one file.
(BZ2)

S36 Dataset ZAP-70 K476-ubiquitinated active simulation N348 Cα—W501 Cα distance file. The distance between the ZAP-70 N348 Cα atom and the W501 Cα atom at each picosecond in the simulations is printed in Angstroms. Data from each of the 32 10-ns simulations is combined into one file.
(BZ2)

S37 Dataset ZAP-70 K476-ubiquitinated inactive simulation N348 Cα—W501 Cα distance file. The distance between the ZAP-70 N348 Cα atom and the W501 Cα atom at each picosecond in the simulations is printed in Angstroms. Data from each of the 32 10-ns simulations is combined into one file.
(BZ2)

S38 Dataset ZAP-70 K377-acetylated active simulation N348 Cα—W501 Cα distance file. The distance between the ZAP-70 N348 Cα atom and the W501 Cα atom at each picosecond in the simulations is printed in Angstroms. Data from each of the 32 10-ns simulations is combined into one file.
(BZ2)

S39 Dataset ZAP-70 K476-acetylated inactive simulation N348 Cα—W501 Cα distance file. The distance between the ZAP-70 N348 Cα atom and the W501 Cα atom at each picosecond in the simulations is printed in Angstroms. Data from each of the 32 10-ns simulations is combined into one file.
(BZ2)

S40 Dataset ZAP-70 K377-Ig domain active simulation N348 Cα—W501 Cα distance file. The distance between the ZAP-70 N348 Cα atom and the W501 Cα atom at each picosecond in the simulations is printed in Angstroms. Data from each of the 32 10-ns simulations is combined into one file.
(BZ2)

S41 Dataset ZAP-70 100-ns active simulation N348 Cα—W501 Cα distance file. The distance between the ZAP-70 N348 Cα atom and the W501 Cα atom at each picosecond in the simulations is printed in Angstroms.
(BZ2)

S42 Dataset ZAP-70 100-ns inactive simulation N348 Cα—W501 Cα distance file. The distance between the ZAP-70 N348 Cα atom and the W501 Cα atom at each picosecond in the simulations is printed in Angstroms.
(BZ2)

S43 Dataset ZAP-70 560-ns K377-ubiquitinated active simulation N348 Cα—W501 Cα distance file. The distance between the ZAP-70 N348 Cα atom and the W501 Cα atom at each picosecond in the simulations is printed in Angstroms.
(BZ2)

S44 Dataset ZAP-70 560-ns K476-ubiquitinated inactive simulation N348 Cα—W501 Cα distance file. The distance between the ZAP-70 N348 Cα atom and the W501 Cα atom at each picosecond in the simulations is printed in Angstroms.
(BZ2)

S45 Dataset ZAP-70 K377-ubiquitinated active simulation kinase-ubiquitin contact matrix file. The fraction of the simulation snapshots where each pair of residues on ZAP-70 and ubiquitin are within 10 Angstroms center-of-mass distances is printed in a matrix.
(BZ2)

S46 Dataset ZAP-70 K377-ubiquitinated inactive simulation kinase-ubiquitin contact matrix file. The fraction of the simulation snapshots where each pair of residues on ZAP-70 and ubiquitin are within 10 Angstroms center-of-mass distances is printed in a matrix.
(BZ2)

S47 Dataset ZAP-70 K476-ubiquitinated active simulation kinase-ubiquitin contact matrix file. The fraction of the simulation snapshots where each pair of residues on ZAP-70 and ubiquitin are within 10 Angstroms center-of-mass distances is printed in a matrix.
(BZ2)

S48 Dataset ZAP-70 K476-ubiquitinated inactive simulation kinase-ubiquitin contact matrix file. The fraction of the simulation snapshots where each pair of residues on ZAP-70 and ubiquitin are within 10 Angstroms center-of-mass distances is printed in a matrix.
(BZ2)

S49 Dataset ZAP-70 K377-Ig domain active simulation kinase-ubiquitin contact matrix file. The fraction of the simulation snapshots where each pair of residues on ZAP-70 and the Ig domain are within 10 Angstroms center-of-mass distances is printed in a matrix.
(BZ2)

S50 Dataset ZAP-70 active simulation kinase domain solvent accessible surface area file. The solvent accessible surface area of the ZAP-70 kinase domain at each picosecond in the simulations is printed. Data from each of the 32 10-ns simulations is combined into one file.
(BZ2)

S51 Dataset ZAP-70 inactive simulation kinase domain solvent accessible surface area file. The solvent accessible surface area of the ZAP-70 kinase domain at each picosecond in the simulations is printed. Data from each of the 32 10-ns simulations is combined into one file.
(BZ2)

S52 Dataset ZAP-70 K377-ubiquitinated active simulation kinase domain solvent accessible surface area file. The solvent accessible surface area of the ZAP-70 kinase domain at each picosecond in the simulations is printed. Data from each of the 32 10-ns simulations is combined into one file.
(BZ2)

S53 Dataset ZAP-70 K377-ubiquitinated active simulation ubiquitin moiety solvent accessible surface area file. The solvent accessible surface area of the ubiquitin moiety at each picosecond in the simulations is printed. Data from each of the 32 10-ns simulations is combined into one file.
(BZ2)

S54 Dataset ZAP-70 K377-ubiquitinated active simulation total solvent accessible surface area file. The solvent accessible surface area of the entire ubiquitinated ZAP-70 at each picosecond in the simulations is printed. Data from each of the 32 10-ns simulations is combined into one file.
(BZ2)

S55 Dataset ZAP-70 K377-ubiquitinated inactive simulation kinase domain solvent accessible surface area file. The solvent accessible surface area of the ZAP-70 kinase domain at each picosecond in the simulations is printed. Data from each of the 32 10-ns simulations is combined into one file.
(BZ2)

S56 Dataset ZAP-70 K377-ubiquitinated inactive simulation ubiquitin moiety solvent accessible surface area file. The solvent accessible surface area of the ubiquitin moiety at each picosecond in the simulations is printed. Data from each of the 32 10-ns simulations is combined into one file.
(BZ2)

S57 Dataset ZAP-70 K377-ubiquitinated inactive simulation total solvent accessible surface area file. The solvent accessible surface area of the entire ubiquitinated ZAP-70 at each picosecond in the simulations is printed. Data from each of the 32 10-ns simulations is combined into one file.
(BZ2)

S58 Dataset ZAP-70 K476-ubiquitinated active simulation kinase domain solvent accessible surface area file. The solvent accessible surface area of the ZAP-70 kinase domain at each picosecond in the simulations is printed. Data from each of the 32 10-ns simulations is combined into one file.
(BZ2)

S59 Dataset ZAP-70 K476-ubiquitinated active simulation ubiquitin moiety solvent accessible surface area file. The solvent accessible surface area of the ubiquitin moiety at each picosecond in the simulations is printed. Data from each of the 32 10-ns simulations is combined into one file.
(BZ2)

S60 Dataset ZAP-70 K476-ubiquitinated active simulation total solvent accessible surface area file. The solvent accessible surface area of the entire ubiquitinated ZAP-70 at each picosecond in the simulations is printed. Data from each of the 32 10-ns simulations is combined into one file.
(BZ2)

S61 Dataset ZAP-70 K476-ubiquitinated inactive simulation kinase domain solvent accessible surface area file. The solvent accessible surface area of the ZAP-70 kinase domain at each picosecond in the simulations is printed. Data from each of the 32 10-ns simulations is combined into one file.
(BZ2)

S62 Dataset ZAP-70 K476-ubiquitinated inactive simulation ubiquitin moiety solvent accessible surface area file. The solvent accessible surface area of the ubiquitin moiety at each picosecond in the simulations is printed. Data from each of the 32 10-ns simulations is combined into one file.
(BZ2)

S63 Dataset ZAP-70 K476-ubiquitinated inactive simulation total solvent accessible surface area file. The solvent accessible surface area of the entire ubiquitinated ZAP-70 at each picosecond in the simulations is printed. Data from each of the 32 10-ns simulations is combined into one file.
(BZ2)

S1 File UBK library file. Ubiquitinated lysine library file for input into *tleap*.
(LIB)

S2 File ZAP-70 active structure Amber parameter file. Amber parameter file output from tleap, used for running Amber simulations.
(BZ2)

S3 File ZAP-70 active structure Amber starting structure file. Amber starting structure file output from tleap, created from ZAP-70 pdb file.
(BZ2)

S4 File ZAP-70 inactive structure Amber parameter file. Amber parameter file output from tleap, used for running Amber simulations.
(BZ2)

S5 File ZAP-70 inactive structure Amber starting structure file. Amber starting structure file output from tleap, created from ZAP-70 pdb file.
(BZ2)

S6 File ZAP-70 K377-ubiquitinated active structure Amber parameter file. Amber parameter file output from tleap, used for running Amber simulations.
(BZ2)

S7 File ZAP-70 K377-ubiquitinated active structure Amber starting structure file. Amber starting structure file output from tleap, created from ZAP-70 pdb file.
(BZ2)

S8 File ZAP-70 K377-ubiquitinated inactive structure Amber parameter file. Amber parameter file output from tleap, used for running Amber simulations.
(BZ2)

S9 File ZAP-70 K377-ubiquitinated inactive structure Amber starting structure file. (BZ2)

S10 File ZAP-70 K476-ubiquitinated active structure Amber parameter file. Amber parameter file output from tleap, used for running Amber simulations.
(BZ2)

S11 File ZAP-70 K476-ubiquitinated active structure Amber starting structure file. Amber starting structure file output from tleap, created from ZAP-70 pdb file.
(BZ2)

S12 File ZAP-70 K476-ubiquitinated inactive structure Amber parameter file. Amber parameter file output from tleap, used for running Amber simulations.
(BZ2)

S13 File ZAP-70 K476-ubiquitinated inactive structure Amber starting structure file. Amber starting structure file output from tleap, created from ZAP-70 pdb file.
(BZ2)

S14 File ZAP-70 K377-acetylated active structure Amber parameter file. Amber parameter file output from tleap, used for running Amber simulations.
(BZ2)

S15 File ZAP-70 K377-acetylated active structure Amber starting structure file. Amber starting structure file output from tleap, created from ZAP-70 pdb file.
(BZ2)

S16 File ZAP-70 K476-acetylated inactive structure Amber parameter file. Amber parameter file output from tleap, used for running Amber simulations.
(BZ2)

S17 File ZAP-70 K476-acetylated inactive structure Amber starting structure file. (BZ2)

S18 File ZAP-70 K377-Ig domain active structure Amber parameter file. Amber parameter file output from tleap, used for running Amber simulations.
(BZ2)

S19 File ZAP-70 K377-Ig domain active structure Amber starting structure file. Amber starting structure file output from tleap, created from ZAP-70 pdb file.
(BZ2)

S20 File Example ZAP-70 cpptraj trajectory analysis script. This is the cpptraj analysis script for the active structure. The analysis commands for each of the 9 ZAP-70 constructs were the same.
(PTRAJ)

S21 File Example ZAP-70 ptraj trajectory distance matrix analysis script. This is one of the ptraj scripts to calculate distance matrices to create a kinase-ubiquitin contact map for the K377-ubiquitinated active structure. The rest of the trajectory was analyzed in additional scripts. The analysis commands for each of the four ubiquitinated ZAP-70 constructs were the same.
(PTRAJ)
