# Supplementary material for: Non-degradative Ubiquitination of Protein Kinases
Source: PLoS Comput Biol. 2016 Jun 2;12(6):e1004898. doi: 10.1371/journal.pcbi.1004898 (PMC4890936; doi:10.1371/journal.pcbi.1004898)
Supplement: S1 Table — (PDF) [file pcbi.1004898.s001.pdf]

S1 Table. Details of simulation solvent box for ZAP-70 constructs.

Construct	# of water molecules in box	Box dimensions (Å)		
Active-state control	12,588	72	78	75
Active-state K377-ubiquitinated	35,867	102	104	108
Active-state K476-ubiquitinated	15,996	88	82	76
Active-state K377-acetylated	12,581	73	79	77
Active-state K377-Ig domain	75,547	130	134	136
Inactive-state control	13,029	75	77	75
Inactive-state K377-ubiquitinated	38,834	106	109	107
Inactive-state K476-ubiquitinated	15,691	87	82	76
Inactive-state K476-acetylated	13,027	77	79	77
