# Supplementary material for: Non-degradative Ubiquitination of Protein Kinases
Source: PLoS Comput Biol. 2016 Jun 2;12(6):e1004898. doi: 10.1371/journal.pcbi.1004898 (PMC4890936; doi:10.1371/journal.pcbi.1004898)
Supplement: S2 Table — (PDF) [file pcbi.1004898.s002.pdf]

S2 Table. HIV dependence of ubiquitination sites identified in Jurkat and HEK293 cells.

**Jurkat + PTMfunc**	**HIV dependence**	**Abundance also affected**
**% of proteasome-sensitive ubiquitination sites**	2%	0%
**% of proteasome-insensitive ubiquitination sites**	0%	0%
**% of all ubiquitination sites**	4%	0%
**HEK293**	**HIV dependence**	
**% of proteasome-sensitive ubiquitination sites**	84%	
**% of proteasome-insensitive ubiquitination sites**	44%	
**% of all ubiquitination sites**	76%	
