# Supplementary material for: Non-degradative Ubiquitination of Protein Kinases
Source: PLoS Comput Biol. 2016 Jun 2;12(6):e1004898. doi: 10.1371/journal.pcbi.1004898 (PMC4890936; doi:10.1371/journal.pcbi.1004898)
Supplement: S3 Table — (PDF) [file pcbi.1004898.s003.pdf]

S3 Table. P-value statistics testing the hypothesis that simulation metrics have the same distribution as the unmodified control simulations started from the same ZAP-70 crystal structure. Using the means of each simulation as the data.

**Data are means of each simulation**	**K377-ubiq**	**K476-ubiq**	**K377-acet**	**K476-acet**	**K377-Ig domain**
**Active state F349 Cα - D379 Cβ distance**	1.422×10^{-10}	0.1060	0.6744		0.1031
**Inactive state F349 Cα - D379 Cβ distance**	0.3394	3.124×10^{-14}		3.026×10^{-4}	
**Active state helicity**	2.219×10^{-6}	0.03266	5.277×10^{-4}		0.04564
**Inactive state helicity**	0.3463	0.1031		0.9098	
**Active state N348 Cα - W501 Cα distance**	7.084×10^{-3}	0.7338	0.5798		0.3673
**Inactive state N348 Cα - W501 Cα distance**	6.334×10^{-11}	1.081×10^{-5}		0.7138	
