# Supplementary material for: Non-degradative Ubiquitination of Protein Kinases
Source: PLoS Comput Biol. 2016 Jun 2;12(6):e1004898. doi: 10.1371/journal.pcbi.1004898 (PMC4890936; doi:10.1371/journal.pcbi.1004898)
Supplement: S4 Table — Using the medians of each simulation as the data. (PDF) [file pcbi.1004898.s004.pdf]

S4 Table. P-value statistics testing the hypothesis that simulation metrics have the same distribution as the unmodified control simulations started from the same ZAP-70 crystal structure. Using the medians of each simulation as the data.

**Data are medians of each simulation**	**K377-ubiq**	**K476-ubiq**	**K377-acet**	**K476-acet**	**K377-Ig domain**
**Active state F349 Cα - D379 Cβ distance**	3.787×10^{-8}	0.1616	0.8992		0.1460
**Inactive state F349 Cα - D379 Cβ distance**	0.4192	9.967×10^{-13}		5.277×10^{-4}	
**Active state helicity**	4.394×10^{-6}*	0.09151*	0.04873*		0.1703*
**Inactive state helicity**	0.3879*	0.09907*		0.8886*	
**Active state N348 Cα - W501 Cα distance**	5.479×10^{-3}	0.6647	0.5616		0.3327
**Inactive state N348 Cα - W501 Cα distance**	3.141×10^{-11}	1.902×10^{-5}		0.8614*	

*indicates p-values that could not be computed exactly with the Wilcoxon test in R because there were duplicate values.
