# Supplementary figures and images for: Non-degradative Ubiquitination of Protein Kinases
Source: PLoS Comput Biol. 2016 Jun 2;12(6):e1004898. doi: 10.1371/journal.pcbi.1004898 (PMC4890936; doi:10.1371/journal.pcbi.1004898)

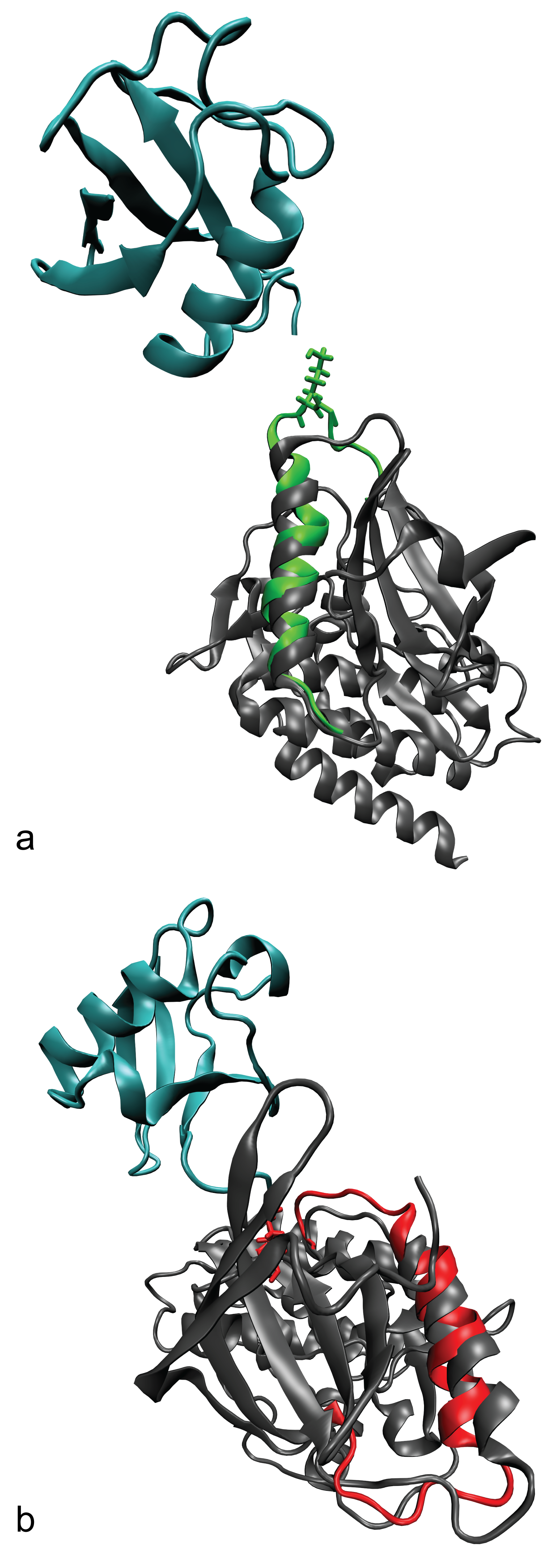

Supplement: S1 Fig — Example ending conformation from active K377-ubiquitinated simulations superimposed on the ZAP-70 active crystal structure (a) and from inactive K476-ubiquitinated simulations superimposed on the ZAP-70 inactive crystal structure (b). The crystal structure is shown in grey, C-helix residues are highlighted in green (a) or red (b), and the ubiquitin molecule is shown in cyan. (a) The F349Cα-D379Cβ distance = 14.9 Å. (b) The F349Cα-D379Cβ distance = 7.6 Å. (TIF) [file pcbi.1004898.s005.tif]

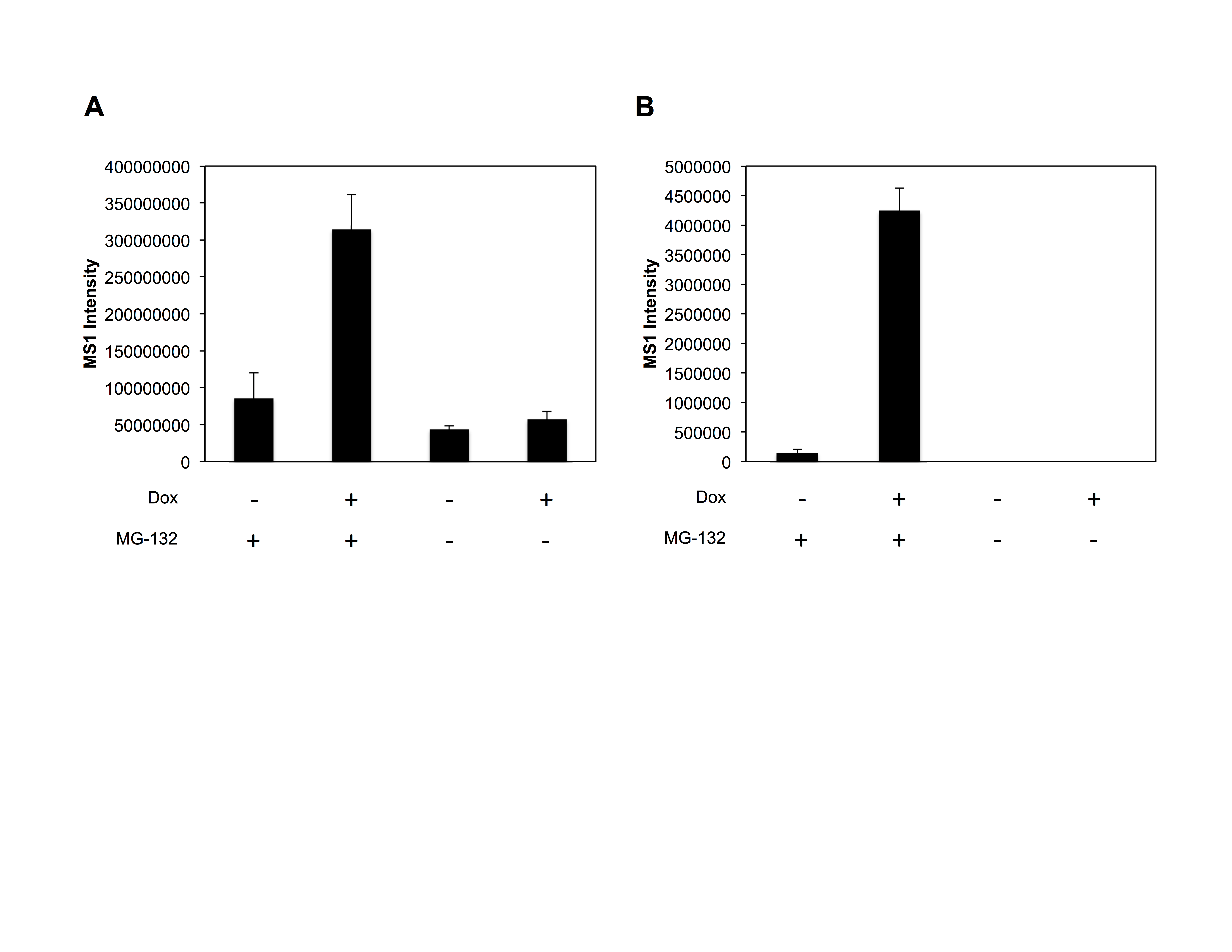

Supplement: S2 Fig — The sum of MS1 intensities of all ubiquitinated peptides are plotted for known substrates of HIV-mediated ubiquitination CD4 (a) and APOBEC3C (b) in response to doxycycline-induced expression of HIV. (TIFF) [file pcbi.1004898.s006.tiff]

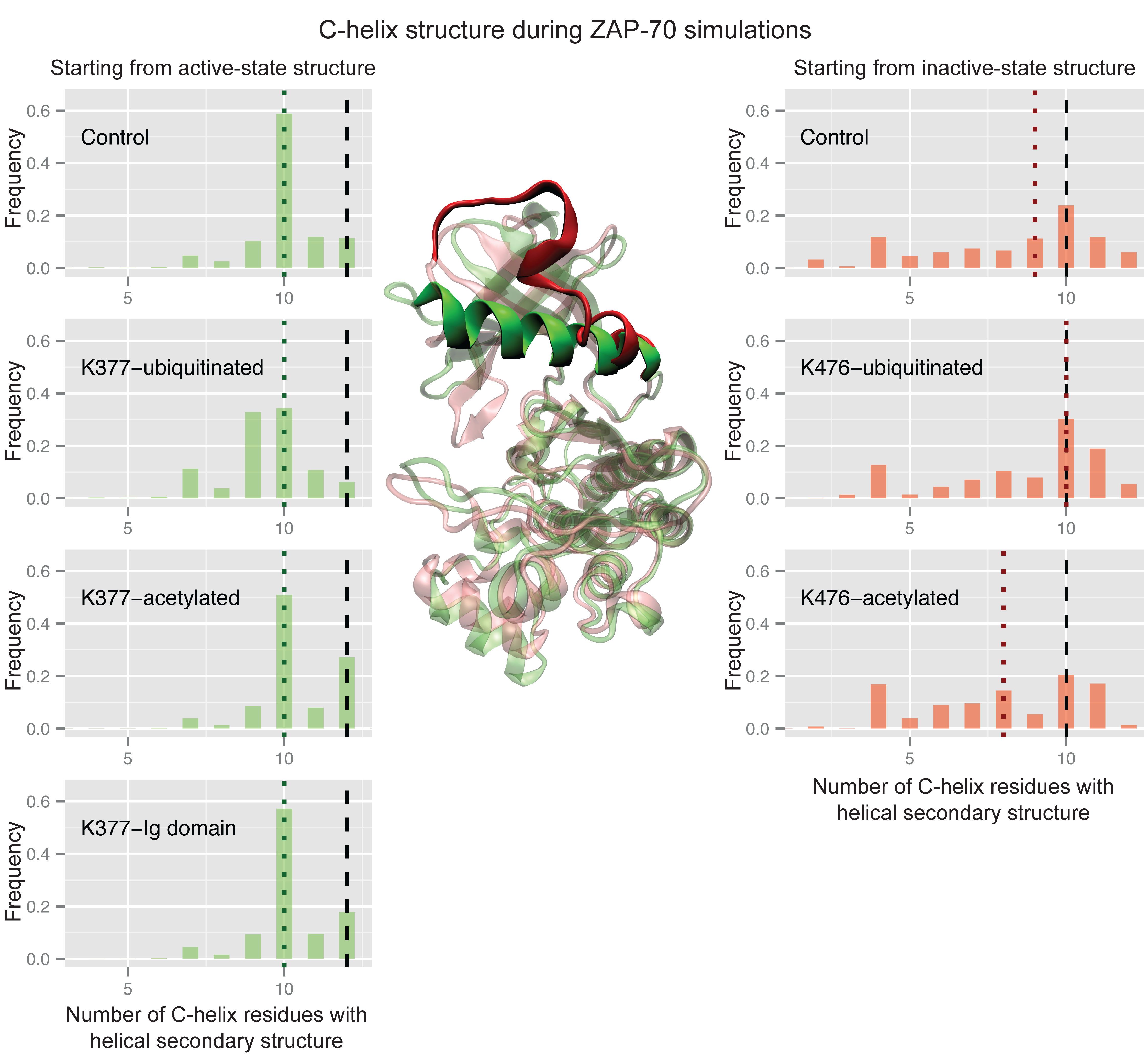

Supplement: S3 Fig — Histograms of the number of residues in the C-helix (380–393) that retain the helical conformation, using the DSSP definition, during ZAP-70 control simulations, K377-monoubiquitinated simulations, K377-acetylated simulations, and simulations with the Ig domain attached at K377, all started from the active state structure (left), as well as control, K476-monoubiquitinated, and K476-acetylated simulations started from the inactive, autoinhibited structure (right). The vertical dashed black lines indicate the distance observed in the starting crystal structure, while the dotted colored lines indicate the median value for each histogram. The histogram is created from combining all structures sampled in each of the 32 independent simulations with snapshots every picosecond. In the center, the C-helix is highlighted on the ZAP-70 active crystal structure (green) and an overlaid structure from the inactive conformation simulations (red) where the C-helix is mostly unraveled (only 6 residues have helical secondary structure). (TIF) [file pcbi.1004898.s007.tif]

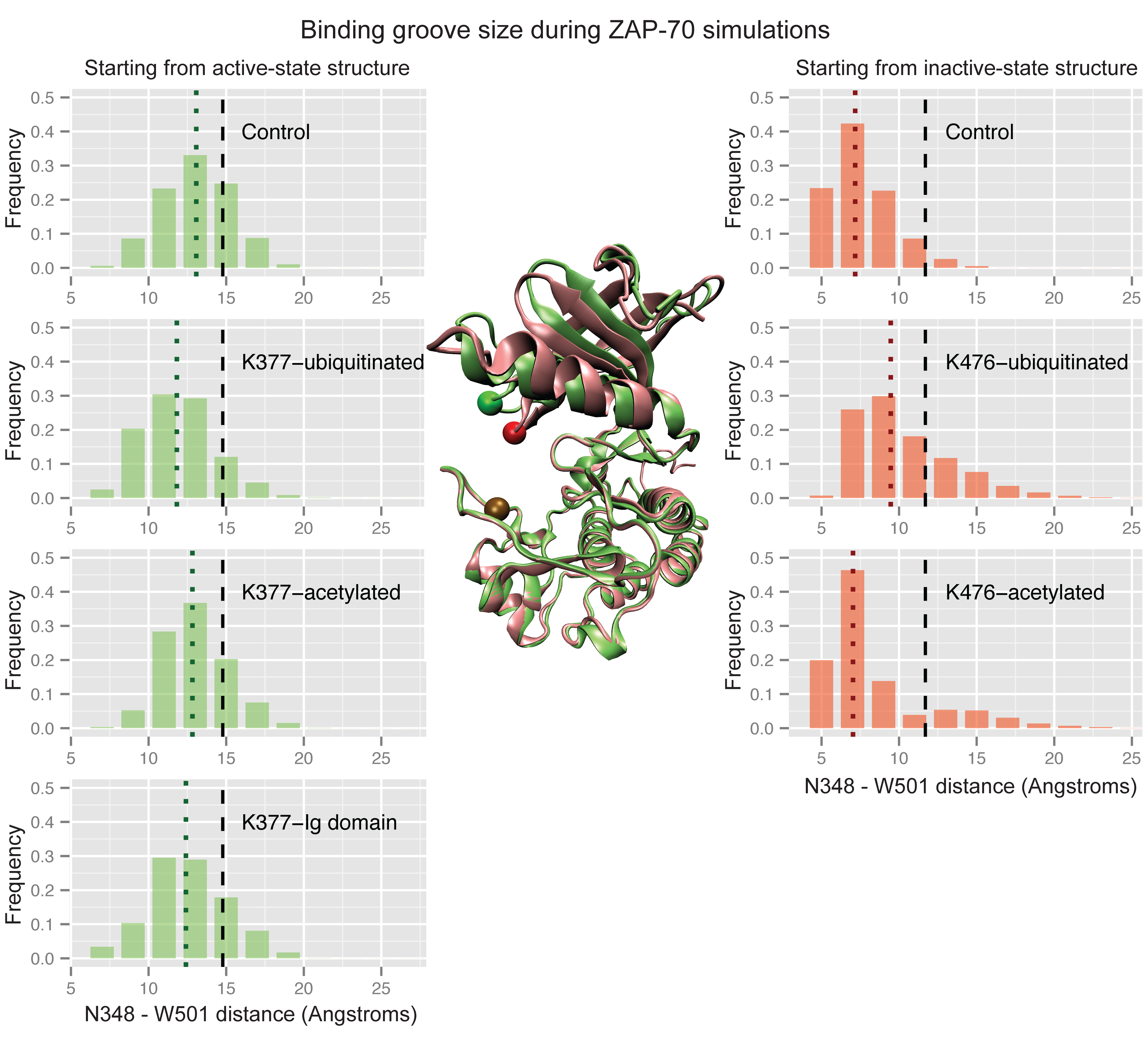

Supplement: S4 Fig — Histograms of the distance between the N348 Cα atom and the W501 Cα atom during ZAP-70 control simulations, K377-monoubiquitinated simulations, K377-acetylated simulations, and simulations with the Ig domain attached at K377, all started from the active state structure (left), as well as control, K476-monoubiquitinated, and K476-acetylated simulations started from the inactive, autoinhibited structure (right). The vertical dashed black lines indicate the distance observed in the starting crystal structure, while the dotted colored lines indicate the median value for each histogram. The histogram is created from combining all structures sampled in each of the 32 independent simulations with snapshots every picosecond. In the center, residues N348 and W501 are highlighted on the overlaid ZAP-70 active (green) and inactive (red) crystal structures. (TIF) [file pcbi.1004898.s008.tif]

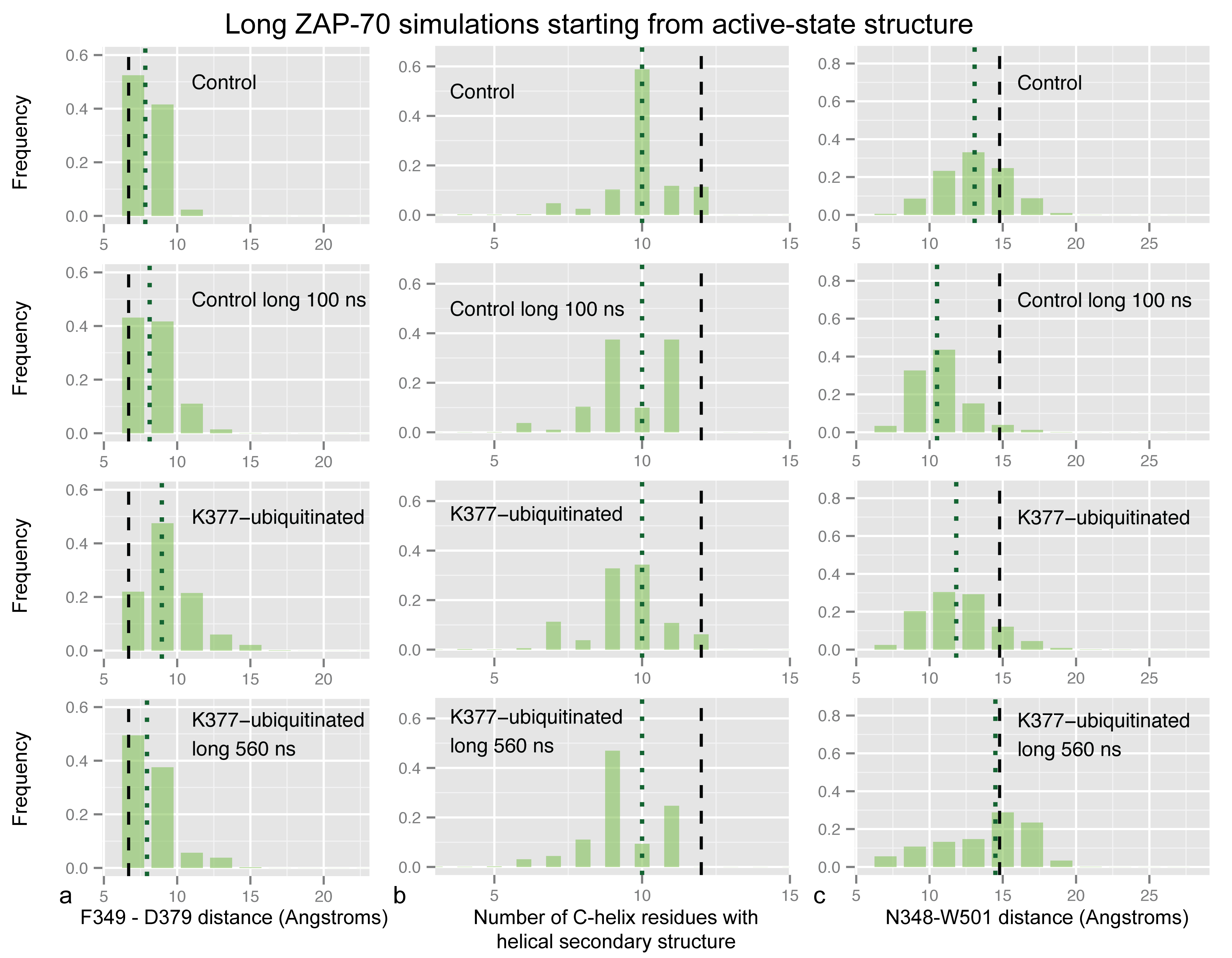

Supplement: S5 Fig — Histograms of the distance between the F349 Cα atom and the D379 Cβ atom (a), the number of residues in the C-helix (380–393) that retain the helical conformation, using the DSSP definition (b), and the distance between the N348 Cα atom and the W501 Cα atom (c). Each of these is plotted for the set of 32 short control simulations, the single 100-ns control simulation, the set of 32 short K377-ubiquitinated simulations, and the single 560-ns K377-ubiquitinated simulation. All of these simulations started from the ZAP-70 active state crystal structure. The vertical dashed black lines indicate the distance observed in the starting crystal structure, while the dotted colored lines indicate the median value for each histogram. The histogram is created by combining all structures sampled in each of the 32 independent simulations (or single long simulation) with snapshots every picosecond. (TIF) [file pcbi.1004898.s009.tif]

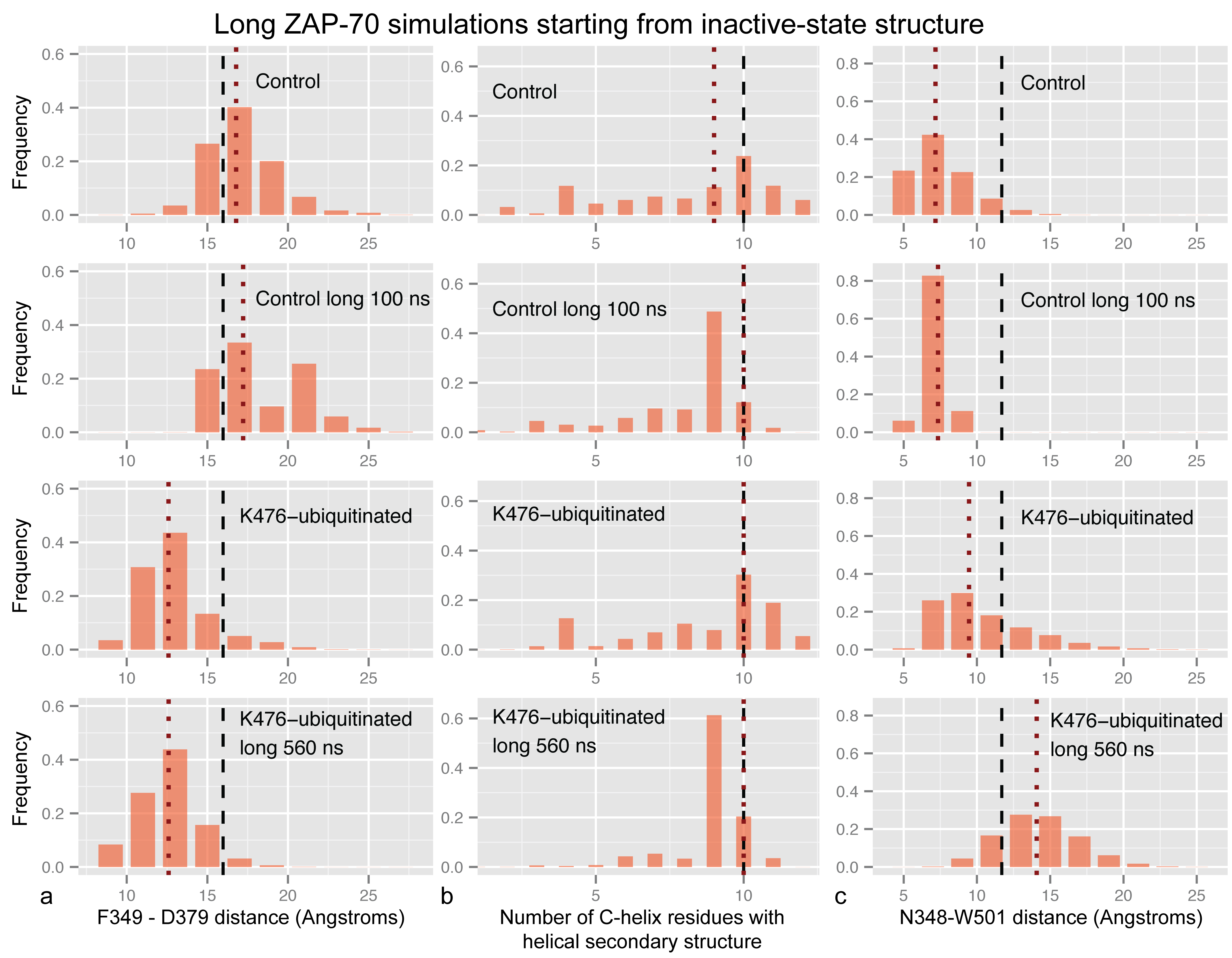

Supplement: S6 Fig — Histograms of the distance between the F349 Cα atom and the D379 Cβ atom (a), the number of residues in the C-helix (380–393) that retain the helical conformation, using the DSSP definition (b), and the distance between the N348 Cα atom and the W501 Cα atom (c). Each of these is plotted for the set of 32 short control simulations, the single 100-ns control simulation, the set of 32 short K476-ubiquitinated simulations, and the single 560-ns K476-ubiquitinated simulation. All of these simulations started from the ZAP-70 inactive state crystal structure. The vertical dashed black lines indicate the distance observed in the starting crystal structure, while the dotted colored lines indicate the median value for each histogram. The histogram is created by combining all structures sampled in each of the 32 independent simulations (or single long simulation) with snapshots every picosecond. (TIF) [file pcbi.1004898.s010.tif]
